# Integrin-Mediated Mechanosensing of Modeled Lymph Node Microenvironment Promotes T Cell Activation via Nuclear Deformation

**DOI:** 10.34133/research.1121

**Published:** 2026-02-06

**Authors:** Jinteng Feng, Guoqing Zhao, Lingzhu Zhao, Luying Geng, Shirong Zhang, Longwen Xu, Mengjie Liu, Guangjian Zhang, Feng Xu, Min Lin, Hui Guo

**Affiliations:** ^1^ Department of Thoracic Surgery, The First Affiliated Hospital of Xi’an Jiaotong University, Xi’an, Shaanxi 710061, P.R. China.; ^2^ The Key Laboratory of Biomedical Information Engineering of Ministry of Education, School of Life Science and Technology, Xi’an Jiaotong University, Xi’an, Shaanxi 710049, P.R. China.; ^3^ Bioinspired Engineering and Biomechanics Center (BEBC), Xi’an Jiaotong University, Xi’an, Shaanxi 710049, P.R. China.; ^4^ Department of Medical Oncology, The Second Affiliated Hospital of Xi’an Jiaotong University, Xi’an, Shaanxi 710004, P.R. China.; ^5^ Department of Medical Oncology, The First Affiliated Hospital of Xi’an Jiaotong University, Xi’an, Shaanxi 710061, P.R. China.; ^6^ The Key Laboratory of Surgical Critical Care and Life Support of Ministry of Education, Xi’an Jiaotong University, Xi’an, Shaanxi, 710049, P.R. China.

## Abstract

Upon tumor metastasis, lymph nodes (LNs) undergo mechanical stiffening, yet how this change influences T cell activation within the microenvironment remains incompletely understood. In particular, the dynamic mechanical forces during activation are transduced by cell–extracellular matrix (ECM) interactions, while cell–cell interactions persist. Here, we established a novel T cell culture platform using hydrogels with tunable stiffness and decoupled presentation of RGD peptide and anti-CD3 monoclonal antibody, separately mimicking ECM–T cell and T cell–antigen-presenting cell interactions. This platform closely mimics the LN microenvironment during T cell activation. By integrating experiments with mathematical modeling, we revealed that T cells sensed mechanical changes in the microenvironment requiring RGD/integrin ligation, while stiff matrix up-regulated F-actin aggregation instead of myosin contraction, deforming the nucleus and promoting yes-associated protein nucleus translocation, resulting in interleukin-2 expression and T cell activation. Our findings shed light on the mechanobiological mechanism underlying the potential benefits of immunotherapy in patients with LN metastases and provide an optimized mechanical platform for studying T cell activation and expansion in vitro.

## Introduction

Solid tumors often engage their draining lymph nodes (LNs), leading to stromal remodeling, tumor metastasis, and immune suppression, resulting in poor prognosis [1,2]. Consequently, treatments such as regional LNs resection, adjuvant postoperative LNs radiotherapy, or prophylactic irradiation of the lymphatic drainage area have become an important part of cancer therapy [3,4]. However, intact LNs are crucial in the era of immunotherapy, ensuring immune reinvigoration and prolonging survival through treatments such as immune checkpoint inhibitors that reactivate exhausted CD8^+^ T cells [5–10]. Since T cell activation primarily occurs in LNs [11], the microenvironment in the LNs markedly influences T cell fate [12,13].

Under pathological conditions such as tumor metastasis, LNs experience marked changes in both biochemical (e.g., cytokine interleukin-2 [IL-2]) [14,15] and biophysical cues (e.g., extracellular matrix [ECM] stiffness) [16–18]. While the role of biochemical cues in regulating T cell activation is well established [19–23], how biophysical cues modulate T cell responses through mechanotransduction, the process by which mechanical stimuli are converted into biochemical signals, remains less understood [24,25]. Accumulating in vitro evidence shows that matrix stiffness is a crucial determinant for T cell activation [16,26–32]. Many prior studies have largely focused on mechanical forces transmitted through the T cell receptor (TCR)–peptide–major histocompatibility complex (p-MHC) bond within the immunological synapse (IS), identifying TCR as a mechanosensitive receptor [33,34]. Complementing the TCR, integrins, which mediate adhesion to ECM and other cells, serve as major mechanosensitive receptors in T cells [35]. Prominent integrins such as α_L_β_2_ (lymphocyte-function-associated antigen-1 [LFA-1]) and α_4_β_1_ (very late antigen-4) markedly influence T cell activation [36–38], and LFA-1–intercellular adhesion molecule-1 interactions at the periphery of the IS contribute to T cell activation and cytotoxicity [39–41]. It remains controversial whether integrin-mediated sensing of matrix stiffness directly regulates nuclear mechanotransduction and downstream activation programs [42,43].

Hydrogel-based platforms (e.g., polyacrylamide, alginate, and polyethylene glycol [PEG]) have been widely used to investigate T cell mechanobiology. However, findings on how matrix stiffness influences T cell activation have been inconsistent [16,27–32], with some studies showing enhanced activation on stiffer substrates and others reporting a biphasic response. We identify 2 major limitations in these prior studies that may contribute to this discrepancy: (a) the use of stiffness ranges that do not fully match the physiological LN microenvironment and (b) the predominant application of mechanical stimulation at the IS, treating the TCR as the primary mechanosensor. In contrast, in vivo T cells migrating through the ECM primarily sense mechanical cues via integrins at the cell–ECM interface [24,44], and this occurs in parallel with T cell–antigen-presenting cell (APC) contacts [45,46]. Hence, our study uniquely decouples stiffness regulation from direct TCR stimulation. By focusing specifically on integrin-mediated mechanotransduction, our platform better mimics the physiological cell–ECM mechanical environment.

To address this, we characterized the stiffness variations and T cell activation in LNs under tumor metastasis in patient samples. We then applied a PEG hydrogel platform that simulates the in vivo stiffness of LNs. Distinct from previous studies that only simulate IS interfaces, we decoupled cyclic Arg–Gly–Asp (cRGD) peptide and anti-CD3 monoclonal antibody presentations to separately mimic the ECM–T cell and T cell–APC interactions. By integrating experiments and mathematical modeling that deconstructs the forces exerted by actin polymerization, we found that a stiff matrix up-regulated F-actin aggregation, leading to T cell activation. Our model showed that, rather than myosin contraction, the vertical forces at the IS derived from actin polymerization are most efficient in inducing nuclear deformation. This deformation promoted the translocation of yes-associated protein (YAP) into the nucleus and the expression of IL-2, resulting in T cell activation. This study provides a theoretical framework from the perspective of mechanobiology for treating metastatic LNs in advanced patients in the era of immunotherapy.

## Results and Discussion

### Metastatic LNs promote matrix stiffening and T cell activation

**Fig. 1.**
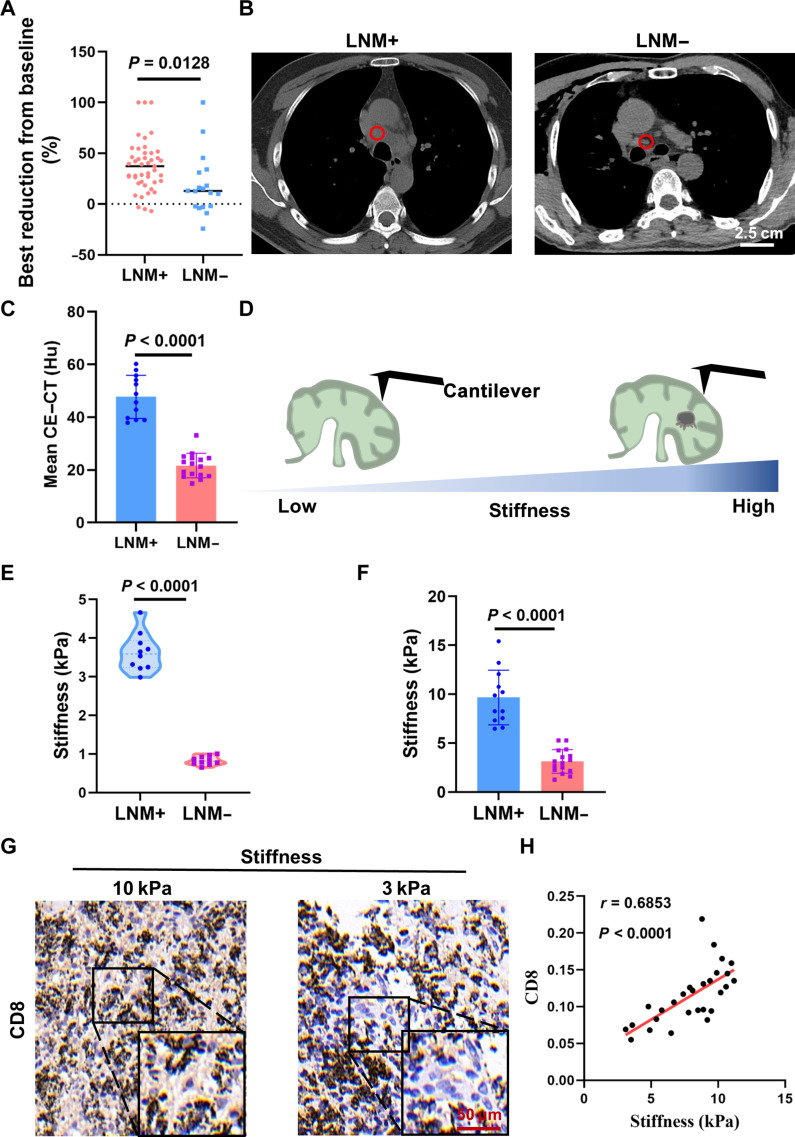
Characterization of the stiffness and the degree of T cell activation within LNs. (A) Comparison of the best reduction from baseline of primary lung cancer under the immunotherapy treatment. A total of 45 patients with tumor-metastasis-positive LN [LNM+] and 18 patients with tumor-metastasis-negative LN [LNM−] were analyzed. (B) Typical CT scan images of patients with lung cancer, the red circle represents the location to measure the mean Hu value of the LN. (C) Corresponding quantification of mean Hu value. CE, contrast-enhanced. (D) Schematic of characterization of LN stiffness using the nanoindenter. LNs were embedded in OCT compound and sectioned into 100-μm-thick slices using a cryostat. The Young’s modulus was calculated by fitting the force–indentation depth curves using the Hertz model. (E) Corresponding quantification of LN stiffness using the nanoindenter, based on the schematic (D). Indentation experiments were performed randomly at the 3 subregions of LNs, *n* > 10. (F) Quantification of LN stiffness using the rotational rheometer. (G) Representative IHC staining images of CD8^+^ cells density in LNs. LNs were categorized into high stiffness (Young’s modulus > 10 kPa) and low stiffness (Young’s modulus < 3 kPa) groups based on the distribution shown in (E) and (F). (H) Correlative analysis between individual LN stiffness values and CD8 expression levels. Each point represents one LN categorized according to the stiffness ranges defined in (G). Data are presented as means ± SEM, and *P* values were obtained by unpaired two-tailed Student’s *t* test (A, C, E, and F) and Mann–Whitney *U* test (H). Scale bars showed on the images.

The impact of LN tumor metastasis status on the effectiveness of immunotherapy, crucial for T cell activation, remains unclear. To investigate this, we conducted a comparative analysis of primary lung cancer in patients who underwent immunotherapy treatment. Our analysis revealed that patients in the LN-metastasis-positive group exhibited greater best reduction from baseline in primary lung cancer size following immunotherapy compared with the LN-metastasis-negative group (Fig. 1A). Tumor metastasis is known to induce substantial remodeling of the immunosuppressive microenvironment in LN, and accumulating evidence also indicates that such remodeling is accompanied by regional LN stiffening, consistent with the surgeon’s palpation during diagnostic or surgical procedures [1,47–49]. Histological examination via hematoxylin and eosin staining further confirmed structural disruption in metastasis-positive LN (Fig. S1A). Subsequently, we used computed tomography (CT) scan to evaluate LN stiffness in vivo, with the Hu value indicating significantly higher stiffness in metastasis-positive LNs compared to metastasis-negative LNs (Fig. 1B and C).

To corroborate the findings of LN stiffness, we analyzed single-cell data of LNs from the Gene Expression Omnibus dataset GSE131907, which includes 10 normal and 7 metastatic LNs [50]. The obtained results demonstrated elevated stiffness scores in tumor-metastasis-positive LNs, as assessed by AddModuleScore, confirming increased stiffness in metastatic LNs (Fig. S2A). Furthermore, in vitro assessments using nanoindentation and a rotational rheometer revealed a 3-fold increase in the average stiffness between metastasis-positive and metastasis-negative LNs (Fig. 1D to F), affirming the stiffening effect of LN metastases.

Gene Ontology enrichment analysis indicated high stiffness-related scores were significantly associated with the pathways related to cell adhesion and T cell activation (Fig. S2B), providing indirect support for altered mechanical regulation. On the basis of the measured Young’s modulus values, LNs were classified into high (>10 kPa) and low (<3 kPa) stiffness groups, consistent with previous reports of LN stiffness ranges at the microscale [51]. These categories were subsequently used to analyze the relationship between stiffness and CD8^+^ T cell activation. We performed immunohistochemistry (IHC) staining and found elevated CD8 expression in metastasis-positive LNs (Fig. 1G and H). To further assess the phenotype of CD8^+^ T cells, we performed multiplex immunofluorescence (IF) staining for CD8, CD69, and CD38, which demonstrated a significantly higher proportion of activated CD8^+^ T cells in LNs with higher stiffness (Fig. S3A). In addition, Masson’s staining revealed markedly increased collagen deposition in metastatic LNs, not only within metastatic tumor regions but also in the surrounding LN stroma (Fig. S3B and C). Together, these results confirm that increased matrix stiffness in metastatic LNs is accompanied by both enhanced CD8^+^ T cell activation and elevated ECM deposition.

### Matrix stiffness regulates T cell activation via integrin-mediated adhesion

**Fig. 2.**
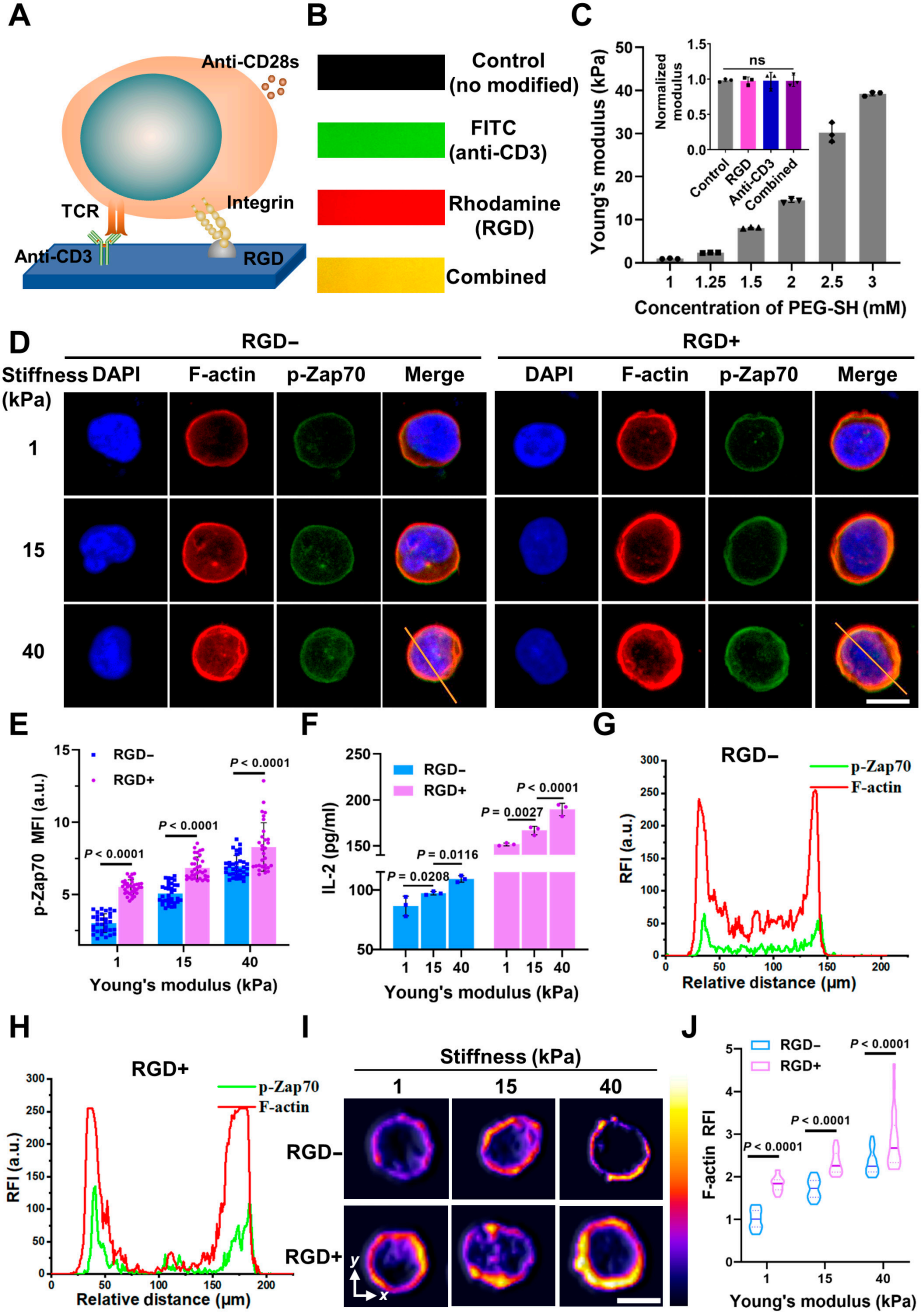
Construction and characterization of a hydrogel system to study the function of integrins in T cells mechanosensing. (A) Schematic illustration of the PEG hydrogels designed for T cell activation. RGD peptide and anti-CD3 monoclonal antibody were modified on PEG hydrogels, which could combine with integrin and TCR, respectively, and soluble anti-CD28 monoclonal antibody was added to the culture medium to provide uniform costimulatory signals, ensuring that variations in T cell activation were primarily due to TCR engagement and substrate stiffness. (B) Fluorescence images displaying the surface distribution of RGD peptides and anti-CD3 antibodies on the PEG hydrogels. The anti-CD3 monoclonal antibody coupled with FITC (green fluorescence), and the RGD peptide coupled with rhodamine (red fluorescence). (C) Quantification of the Young’s modulus of hydrogels synthesized with varying PEG-SH concentrations and different functionalization states: unmodified (control), RGD-only (RGD), anti-CD3-only (anti-CD3), and combined RGD and anti-CD3 (combined). Each point represents an independently synthesized hydrogel. ns, not significant. (D) Representative confocal images of T cells on 1, 15, and 40 kPa of different modified substrates, as DAPI for nucleus (blue), F-actin (red), and p-Zap70 (green). Yellow lines indicated the pixel regions used to generate intensity profiles of F-actin and p-Zap70, which were randomly selected within the cell area. (E) Corresponding quantitative analysis of p-Zap70 for (D). MFI, mean fluorescence intensity. (F) Quantification of IL-2 expression of T cells on 1, 15, and 40 kPa of different modified substrates and comparison of IL-2 expression across different stiffnesses within the same RGD condition. An axis break is introduced on the *y* axis to allow clear visualization of data spanning different expression ranges. (G) Representative colocalization analysis of F-actin and p-Zap70 without RGD modified on the PEG substrates. RFI, relative fluorescence intensity. (H) Representative colocalization analysis of F-actin and p-Zap70 with RGD modified on the PEG substrates. (I) Representative volcano plot illustrating changes in F-actin expression in T cells cultured on hydrogels with stiffnesses of 1, 15, and 40 kPa under different functionalization conditions, which represent fluorescence intensity expressed in arbitrary units (a.u.). (J) Corresponding quantification of F-actin fluorescence intensity for (I). RGD− represents only anti-CD3 monoclonal antibody modified on the substrates, and RGD+ represents both RGD peptide and anti-CD3 monoclonal antibody modified on the substrates. For the quantification of p-Zap70 and F-actin, the number of T cells used for statistics was ≥30. Data are presented as means ± SEM, and *P* values were obtained using one-way ANOVA, followed by Tukey’s post hoc test (C, E, F, and J). Scale bars, 10 μm.

However, because of the complexity of the in vivo microenvironment, a direct causal relationship between matrix stiffness and T cell activation is difficult to establish. Hence, we next sought to determine whether matrix stiffness alone is sufficient to regulate T cell activation under well-controlled in vitro platform. To address this challenge, as soluble anti-CD28 was used to provide uniform costimulatory signals, we utilized a PEG hydrogel modified with RGD peptide and anti-CD3 monoclonal antibody to mimic the ECM–T cell and T cell–APC interaction (Fig. 2A and Fig. S4A). Fluorescence assays confirmed functional conjugation on the surface of the PEG hydrogel (Fig. 2B and Fig. S4B). Independently tunable hydrogel stiffness, ranging from approximately 1 to 40 kPa, was synthesized by varying the final concentrations of PEG thiol (PEG-SH) while maintaining a constant concentration of PEG maleimide (PEG-MAL) (Fig. 2C and Fig. S4C and D), thus encompassing the biophysical spectrum of LNs physiological to tumor metastatic conditions in vivo [46]. Importantly, we confirmed that ligand modification did not affect the stiffness of PEG hydrogels. In addition, hydrogels modified with RGD alone fail to support cell spreading (Fig. S4E and F), suggesting a distinct mechanobiological mechanism for T cells in responding to matrix stiffness compared to most cells [43].

We then explored how matrix stiffness affects T cell activation. To further investigate the early activation signals in T cells responding to substrate stiffness, we assessed the phosphorylation level of zeta-chain-associated protein kinase 70 (p-Zap70). Zap70 is a key proximal signaling molecule downstream of TCR engagement, and its phosphorylation represents an early event during T cell activation, including mechanotransduction processes. Therefore, p-Zap70 serves as a sensitive indicator of stiffness-induced T cell activation. We first determined the temporal dynamics of p-Zap70 within minutes and confirmed the necessity of CD28 costimulatory signal during T cell activation to ensure optimal activation levels (Fig. S5A to C), consistent with previous studies [27,32]. With these conditions in place, we explored the effect of matrix stiffness on T cell activation and observed significantly increased average fluorescence intensity of p-Zap70 with RGD presentation across all stiffness levels (Fig. 2D and E). Furthermore, we confirmed that integrin adhesion promoted T cell activation through phosphorylation of Zap70 rather than altering expression levels (Fig. S5C to E). In addition, IL-2 expression gradually increased with RGD modification at all stiffness levels, further supporting the promotion of T cell activation by matrix stiffness via integrin-mediated adhesion (Fig. 2F). These results confirm the finding in the previous studies that a range of stiffness can promote T cell activation through anti-CD3/TCR binding; however, the most important finding was that in the mechanotransduction of ECM, which has pronounced stiffness variation during T cell activation, integrin-mediated adhesion plays the major role.

Actin cytoskeleton polymerization is crucial in cellular response to matrix stiffness [43]. Colocalization of p-Zap70 with F-actin validated the influence of matrix stiffness on T cell activation, in line with previous reports [26], with more pronounced colocalization observed in the presence of RGD/integrin binding (Fig. 2G and H). Given that Zap70 protein binds to the TCR complex region for phosphorylation, this result implies that integrin adhesion may regulate T cell activation via the cytoskeletal mechanisms. Moreover, the observed increase in relative fluorescence intensity of F-actin under the RGD modification at all stiffness levels (Fig. 2I and J) further supports the notion that integrin adhesion promotes T cell activation, dependent on matrix stiffness and cytoskeletal rearrangement.

In this study, we used Jurkat T cells as a model system to investigate the effects of substrate stiffness on T cell activation, specifically focusing on β_1_-integrin-mediated mechanosensing. T cells are known to express multiple integrin subtypes, including β_1_ and α_v_β_3_, both capable of binding RGD motifs [52–54]. To specifically assess the role of β_1_ integrins, we used a β_1_-integrin-neutralizing antibody (HMb1-1), which significantly reduced CD25 expression levels (Fig. S5F and G). This suggests that although multiple integrins can interact with RGD, β_1_ integrins play a predominant role in mediating stiffness-dependent T cell activation in our system. Although Jurkat cells do not fully recapitulate all aspects of primary T cell biology, they provide a well-controlled and accessible platform for dissecting integrin-mediated signaling pathways in response to matrix stiffness. To further validate our findings, we examined additional activation markers beyond p-Zap70 and IL-2. In primary murine CD8^+^ T cells, we assessed p-Zap70, CD69, CD25, and interferon-γ levels (Fig. S6A to F), while in Jurkat T cells, we detected CD25 expression (Fig. S6G and H). Consistent across both cell types, RGD-modified matrix enhanced the expression of all these activation markers, confirming that the stiffness-dependent activation patterns observed in Jurkat cells were reproducible in primary T cells. These results not only strengthen the physiological relevance of our findings but also support the use of Jurkat T cells as a mechanistic model for investigating integrin-mediated stiffness sensing in T cell activation. Nevertheless, the mechanobiological mechanisms underlying matrix-stiffness-modulated T cell activation need to be further explored.

### Matrix mechanotransduction drives T cell activation through nuclear deformation and YAP nuclear localization

**Fig. 3.**
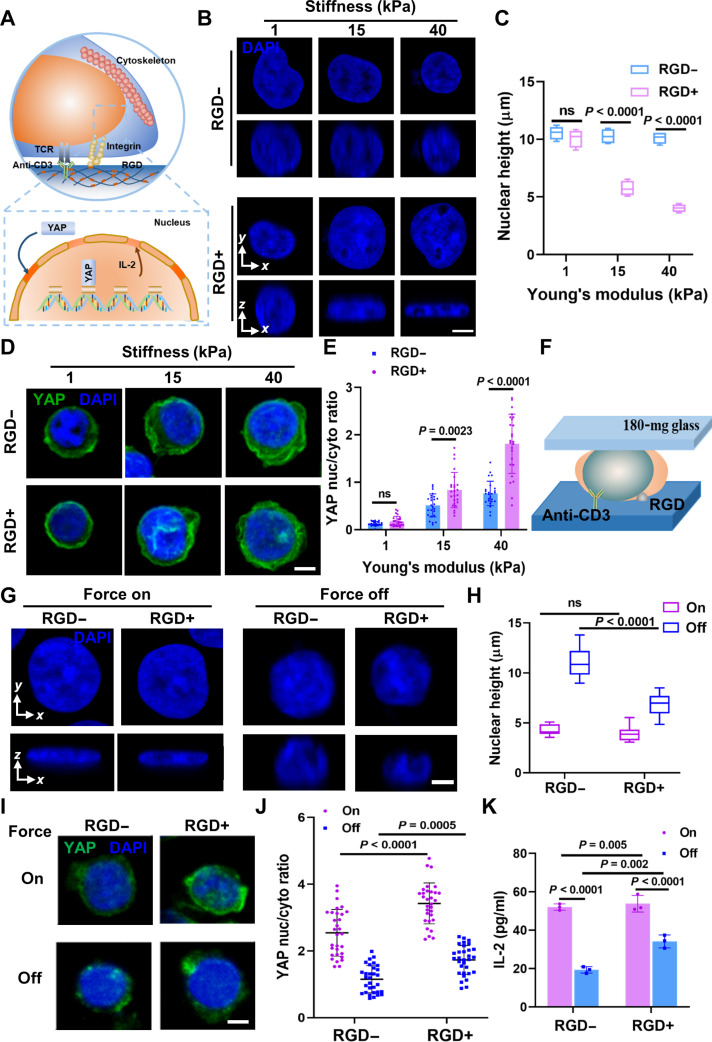
Integrins mediate mechanosensing by inducing nuclear deformation and regulating YAP signaling to modulate T cell activation. (A) Schematic of a possible pathway for integrins to mediate mechanosensing of matrix stiffness to modulate T cell activation. Stiff matrix up-regulated F-actin aggregation mediated by integrins, deformed the nucleus, and thereby promoted the translocation of YAP into the nucleus and expression of IL-2. (B) Representative DAPI staining at the cross-sectional side view was captured along the *xy* and *xz* plane crossing the center of the nucleus on 1, 15, and 40 kPa of different modified substrates. (C) Corresponding quantification of nuclear height for (B). (D) Representative YAP images on 1, 15, and 40 kPa of different modified substrates. (E) Corresponding quantification of YAP nuc/cyto for (D). (F) Schematic of the model for the direct mechanical application to the T cells’ nucleus. A 180-mg slide was added to the whole 15-kPa substrate after inoculation of T cells. (G) Representative DAPI staining at the cross-sectional side view was captured along the *xy* and *xz* plane crossing the center of the nucleus under different mechanical conditions. (H) Corresponding quantification of nuclear height for (G). (I) Representative YAP images under different mechanical conditions. (J) Corresponding quantification of YAP nuc/cyto for (J). (K) Quantification of IL-2 expression under different mechanical conditions. Force on represents the system that applies direct mechanical application on the T cells’ nucleus, and force off represents the system that does not apply direct mechanical application on the T cells’ nucleus. For the quantification of nuclear height and YAP nuc/cyto ratios, the number of T cells used for statistics was ≥30; these cells were selected from over 10 independent fluorescence microscopy images. Data are presented as means ± SEM, and *P* values were obtained using one-way ANOVA, followed by Tukey’s post hoc test (C, E, H, J, and K). Scale bars, 5 μm. Note that the stiffness for all functionalization experiments was fixed at 15 kPa, unless otherwise specified.

In contrast to adherent cells, where the role of YAP nuclear localization in integrin-mediated matrix mechanotransduction is well established [43,55,56], our understanding of YAP nuclear localization in T cell activation [16], particularly regarding its connection to nuclear deformation mediated by integrins, remains limited. We hypothesized that integrin-mediated matrix stiffness induces nuclear deformation by promoting F-actin aggregation, thereby activating T cells (Fig. 3A). To validate this hypothesis, we characterized F-actin, nucleus height, and YAP nucleoplasmic ratios. Remarkably, we observed a progressive decrease in nucleus height with increasing matrix stiffness, particularly in the presence of integrin adhesion, indicative of nuclear flattening along the *z* axis (Fig. 3B and C). This trend correlated with enhanced nucleation of YAP under similar conditions (Fig. 3D and E), suggesting that integrin-mediated matrix mechanics indeed induce nuclear deformation. Besides, while the long-term regulatory role of YAP in T cells may involve restraining effector expansion, YAP nuclear translocation in response to mechanical cues appears to positively contribute to early T cell activation. These results highlight the dynamic and context-dependent role of YAP in T cell mechanotransduction.

To further explore the pivotal roles of nuclear deformation and the associated YAP localization in T cell activation, we investigated whether direct mechanical force applied to the nucleus could enhance T cell activation. We introduced a 180-mg glass slide to the hydrogel system post-T-cell-culture (Fig. 3F), establishing a model to examine the effect of direct force on the nucleus on T cell activation (Fig. S7A). Characterization of nucleus height, YAP nucleoplasm ratios, and IL-2 expression revealed that direct force on T cells resulted in significant nuclear flattening, surpassing the effect induced solely by integrin-mediated adhesion (Fig. 3G to K). Moreover, the changes in YAP nucleoplasm ratio and IL-2 expression correlated with nuclear height alterations (Fig. 3I to K), emphasizing the central role of nuclear deformation in T cell activation. Importantly, this mechanical process facilitated YAP nuclear translocation, consistent with previous studies showing that YAP preferentially localizes to the nucleus in stiff microenvironments, thereby amplifying T cell activation signals [16]. Mechanistically, integrin-mediated adhesion to the stiffened matrix promotes F-actin polymerization and cytoskeletal tension, which, in turn, transmit increased forces toward the nucleus. These forces induce predominantly vertical nuclear deformation, rather than isotropic *x*–*y* deformation, reflecting actin-polymerization-driven force transmission in T cells and thereby enhancing nuclear mechanosensitivity and facilitating YAP nuclear entry. Together, these findings indicate that matrix-stiffness-induced mechanical forces, transmitted via integrin adhesion and nuclear deformation, converge on YAP-dependent transcriptional programs to regulate T cell activation.

**Fig. 4.**
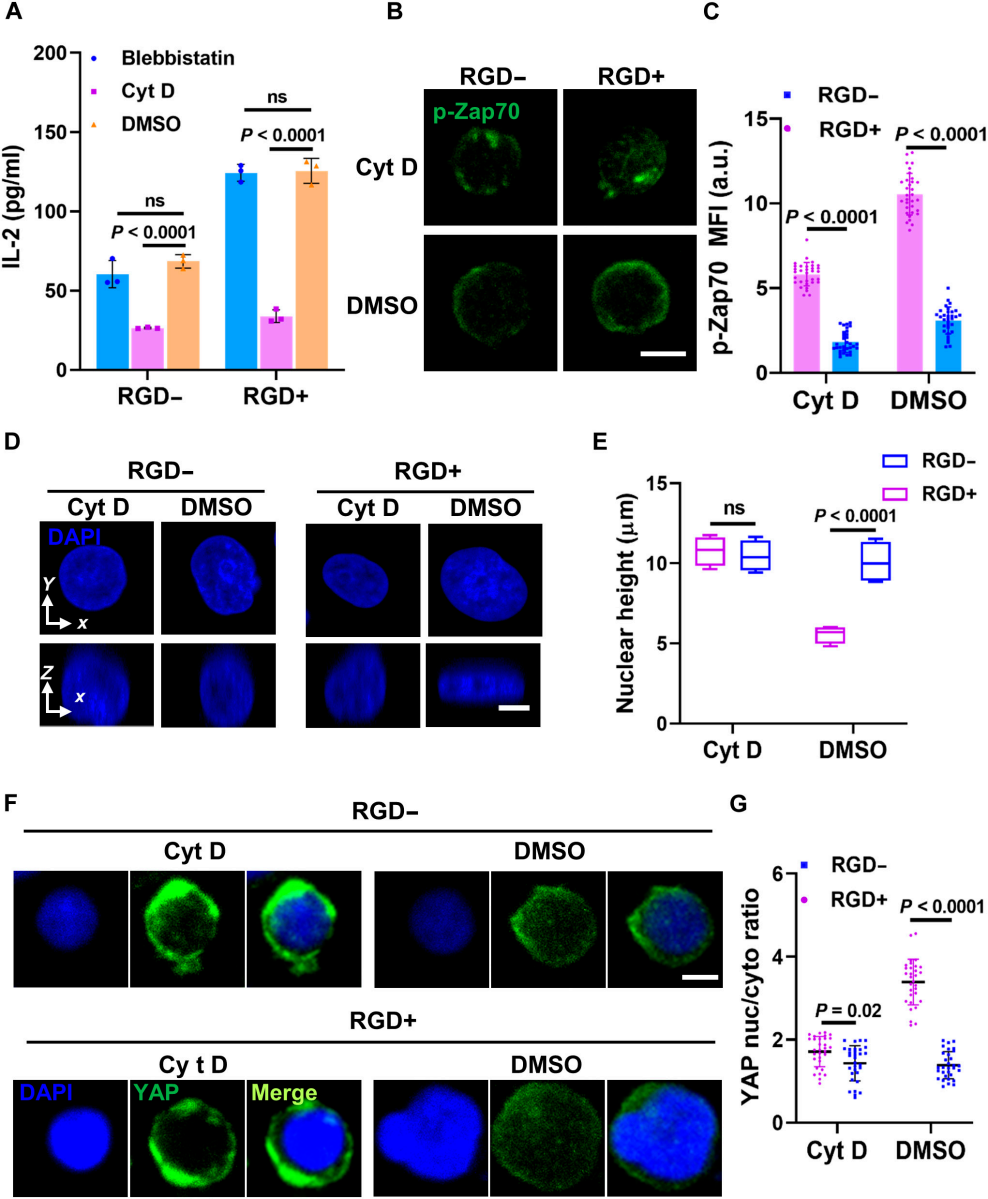
Actin polymerization rather than myosin contraction regulates T cell activation. (A) Quantification of IL-2 expression of T cells which were treated with dimethyl sulfoxide (DMSO; control), Cyt D (actin polymerization inhibitor), or blebbistatin (inhibitor of myosin) on different modified substrates. (B) Representative p-Zap70 images of T cells, which were treated with DMSO or Cyt D on different modified substrates. (C) Corresponding quantification of p-Zap70 for (B). (D) Representative DAPI staining at the cross-sectional side view was captured along the *xy* and *xz* plane crossing the center of T cells’ nucleus, which were treated with DMSO or Cyt D on different modified substrates. (E) Corresponding quantification of nuclear height for (D). (F) Representative YAP images of T cells that were treated with DMSO or Cyt D on different modified substrates. (G) Corresponding quantification of YAP nuc/cyto for (F). All images were acquired and processed under identical conditions. For the quantification of nuclear height and YAP nuc/cyto ratios, the number of T cells used for statistics was ≥30; these cells were selected from over 10 independent fluorescence microscopy images. Data are presented as means ± SEM, and *P* values were obtained using one-way ANOVA, followed by Tukey’s post hoc test (A, C, E, and G). Scale bars, 10 μm (B) and 5 μm (D and F).

To further confirm these findings, we intervened in the cytoskeleton and YAP. While the actin polymerization inhibitor cytochalasin D (Cyt D) significantly inhibited IL-2 expression, the myosin II adenosine triphosphatase inhibitor blebbistatin had a negligible effect, suggesting that actin polymerization, rather than myosin contraction, plays a central role in stiffness-mediated T cell activation (Fig. 4A). This observation is consistent with previous reports suggesting that force generation in T cells relies primarily on actin polymerization [27,57]. Further supporting the role of actin polymerization in T cell activation, Cyt D abolished differences in expression of p-Zap70, nucleus height, and YAP nucleoplasmic ratio induced by integrin adhesion (Fig. 4B to G), suggesting that integrin-mediated actin remodeling is indispensable for the mechanotransductive cascade linking matrix stiffness to T cell activation. In addition, small-interfering-RNA-mediated YAP knockdown abolished the stiffness- and RGD-dependent differences in IL-2 expression, affirming the essential role of YAP in mechanosensitive T cell activation (Fig. S8A and B). Consistent with previous findings by Meng et al. [16], this suggests that YAP primarily regulates activation-related signaling rather than general cell survival or growth. To further probe the causal relationship between nuclear deformation and YAP nuclear localization, we used the nuclear export inhibitor leptomycin B (LMB). IF staining revealed that YAP remained nuclear in both control and RGD-modified groups under LMB treatment, while CD25 fluorescence intensity showed no significant differences between groups (Fig. S8C to E).

Together, these results elucidate the mechanobiological mechanism whereby integrin-mediated matrix stiffness promotes T cell activation by facilitating actin-polymerization-induced nuclear deformation and YAP nuclear localization. However, further validation of this conclusion through investigating the specific role of actin polymerization in nuclear deformation is warranted.

### Matrix-stiffness-driven T cell activation depends primarily on actin polymerization rather than myosin contraction

As previously discussed, matrix stiffness triggers forces on the nucleus of T cells via integrin-mediated adhesion, leading to nuclear deformation and subsequent T cell activation (Fig. 3). Building on this understanding, we then asked whether myosin contraction in T cells contributes to nuclear deformation as has been demonstrated in most cells [58–60]. This was falsified by the observation that inhibiting myosin contraction did not eliminate the difference in IL-2 expression (Fig. 4A), aligning with some existing reports [57,61], while conflicting with others [62,63]. This suggests that myosin-driven contractile force may not be the primary driver of nuclear deformation during T cell activation.

**Fig. 5.**
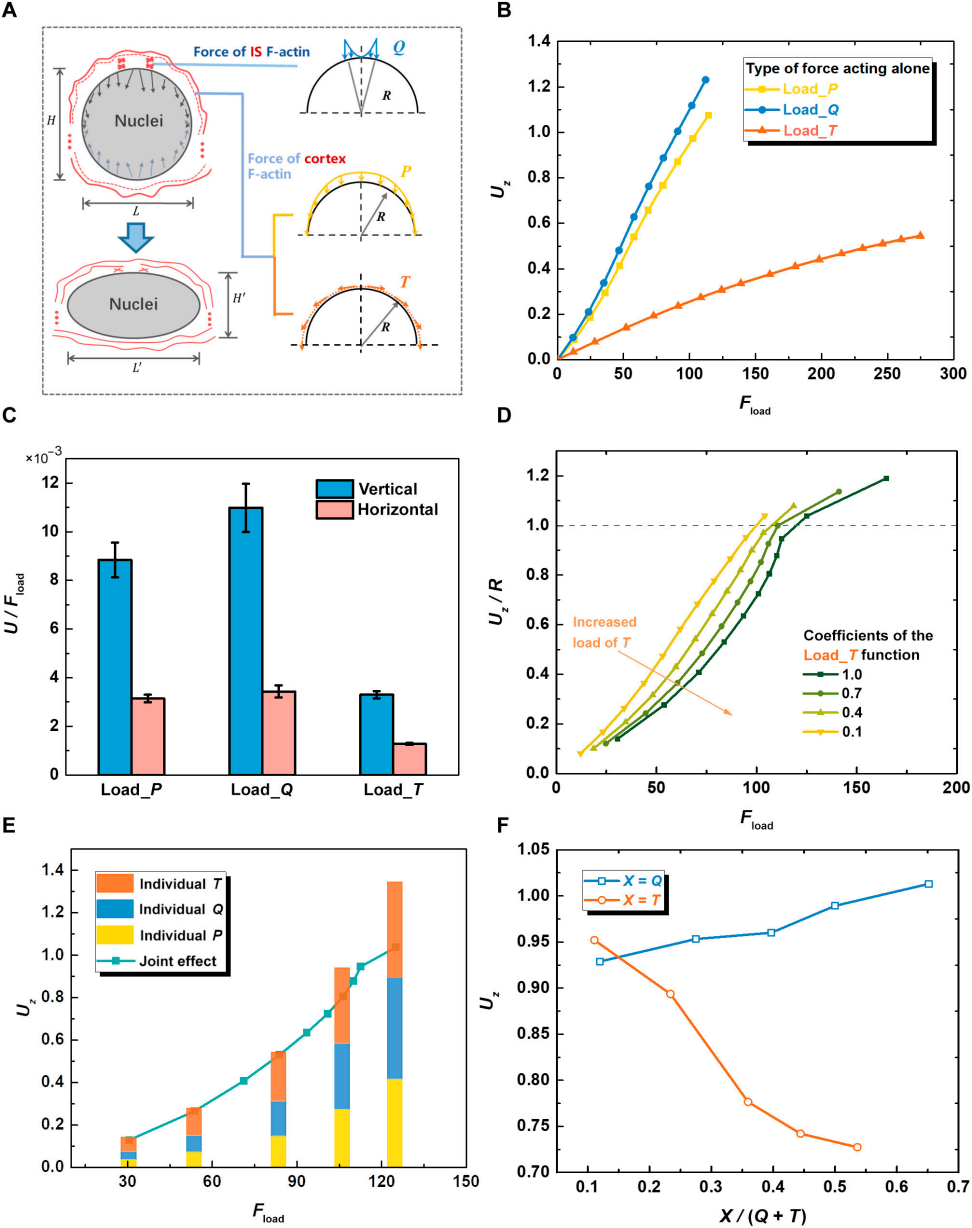
A theoretical model to qualitatively analyze the force characteristics for nuclear deformation during T cell activation. (A) Schematic of the force model for nuclear deformation in activated T cells. Left: The shape of the nucleus gradually flattened. The red solid lines indicated the cortical actin, the red dashed lines indicated the actin filaments undergoing polymerization, and the shape “X” indicated the actin filaments under the IS. Right: The force originating from actin polymerization was divided into 3 distributed loads. The force on the cortical actin layer of the T cell was divided into the vertical distribution load *P* and the tangential distribution load *T*, and the force exerted by the actin in the central IS was simplified as the vertical load *Q*. All the data have already been dimensionless. (B) The relationships of nuclear deformation in the vertical direction versus the magnitude of the load when each of the 3 forces is loaded individually. (C) The nuclear deformation per unit of force when each of the 3 forces is loaded individually. (D) The vertical deformation of the nucleus versus the load under different coefficients (determining the magnitude) of the load *T* function. (E) The deformation in the vertical direction versus the corresponding magnitudes of *P*, *Q*, and *T* acting individually in this load combination using the curve with a coefficient of 1.0 in (D) as an example. (F) The relationships of vertical nuclear deformation versus the proportional contribution of load *Q* or *T* when the total load was constant.

Motivated by the observations that actin polymerization facilitates *Listeria* moves rapidly through the host cytoplasm [64], cellular pseudopod extension [65], and fibroblast spreading [66]. We thus explored whether remodeling of the T cell cytoskeleton leads to nuclear compression from both the thickened actin layer and the actin aggregated at the IS. On the basis of these observations, we proposed a qualitative model to analyze how actin polymerization forces contribute to nuclear deformation during T cell activation. We hypothesized that the thickened cortical actin layer surrounding the nucleus [67,68], along with actin at the circumferential/distal supramolecular activation cluster in the IS [69], would compress the nucleus, consequently resulting in nuclear deformation (Fig. 5A).

To facilitate analysis, we model the compression of the nucleus by the thickened cortical actin layer as a vertical force *P* and a tangential force *T* and the actin-mediated propulsion at the IS as a vertical force *Q* (Fig. 5A). The modeling and subsequent data normalization methods can be found in Text S1 and Table S1. Initially, we assigned the *P*, *Q*, and *T* loads using trigonometric and linear functions to simulate load distribution. The simulation results indicate that different configurations of *P*, *Q*, and *T* loads minimally affect the deformation–force curve of the nucleus (Fig. S9A to C). Applying vertical loads *P* and *Q* individually produced comparable nuclear deformation patterns, where tangential force *T* resulted in less deformation, indicating that vertical forces dominate nuclear flattening in this system (Fig. 5B). These simulated results are consistent with experimental observations (Fig. 3B), showing a significant decrease in nuclear height and elongation along its long axis (Fig. S9D and E). Quantitative analysis showed that vertical forces, particularly force *Q* at the synaptic site, exhibited the highest efficiency in nuclear deformation (Fig. 5C). This finding aligns with previous studies, suggesting the substantial impact of actin polymerization at the IS on nuclear morphology [70].

To examine the practical implications of force on the T cell nucleus, we combined 3 types of loads to generate 4 load curves (Fig. 5D). It is worth noting that increasing the proportion of tangential force *T* will reduce the longitudinal compression of the nucleus (below the dashed line). In addition, the actual nuclear deformation is always less than or equal to the sum of displacements caused by the individual actions of *P*, *Q*, and *T* (Fig. 5E). This suggests that the tangential force *T* indeed attenuates the longitudinal compression of the nucleus (Fig. S9E), maintaining nuclear morphology (Fig. 3B and C). In addition, vertical forces have a marked impact on nucleus deformation when the total load is fixed (Fig. 5F). Specifically, when the cortex forces *P* and *T* were fixed, the IS force *Q* could produce greater vertical deformation and strain energy of the nucleus (Fig. S9E to G). This suggests that T cells may efficiently regulate nucleus deformation by adjusting forces when the total actin pool is limited. The qualitative trend is captured by repurposing Yi’s 200-nm thickening increments as vertical loads [71], adopting Yang’s actin-related protein 2/3 expansion without myosin [72], and using Fabrikant’s measured nuclear compliance to set the strain scale [70].

Together, our combined simulations and experimental data demonstrate that actin polymerization alone, without involvement of myosin, is sufficient to induce nuclear deformation in T cells, in contrast to what has been reported for adherent cells [60,73]. In this process, *P* sets the initial thrust for deformation, *Q* provides the most efficient gain, and *T* buffers against overflattening (Fig. 5C and F and Fig. S9F). While this model has quantitative limitations and does not capture the potential contributions of microtubules, lamin A, or force pulses, it offers a possible mechanistic framework through which actin forces at the IS may play a critical role in nuclear deformation. Together, these insights suggest that cells may fine-tune the distribution of actin-generated forces to modulate nuclear mechanics and downstream transcriptional programs in response to matrix stiffness.

### Integrin-mediated matrix stiffness sensing drives T cell activation independently of the IS

**Fig. 6.**
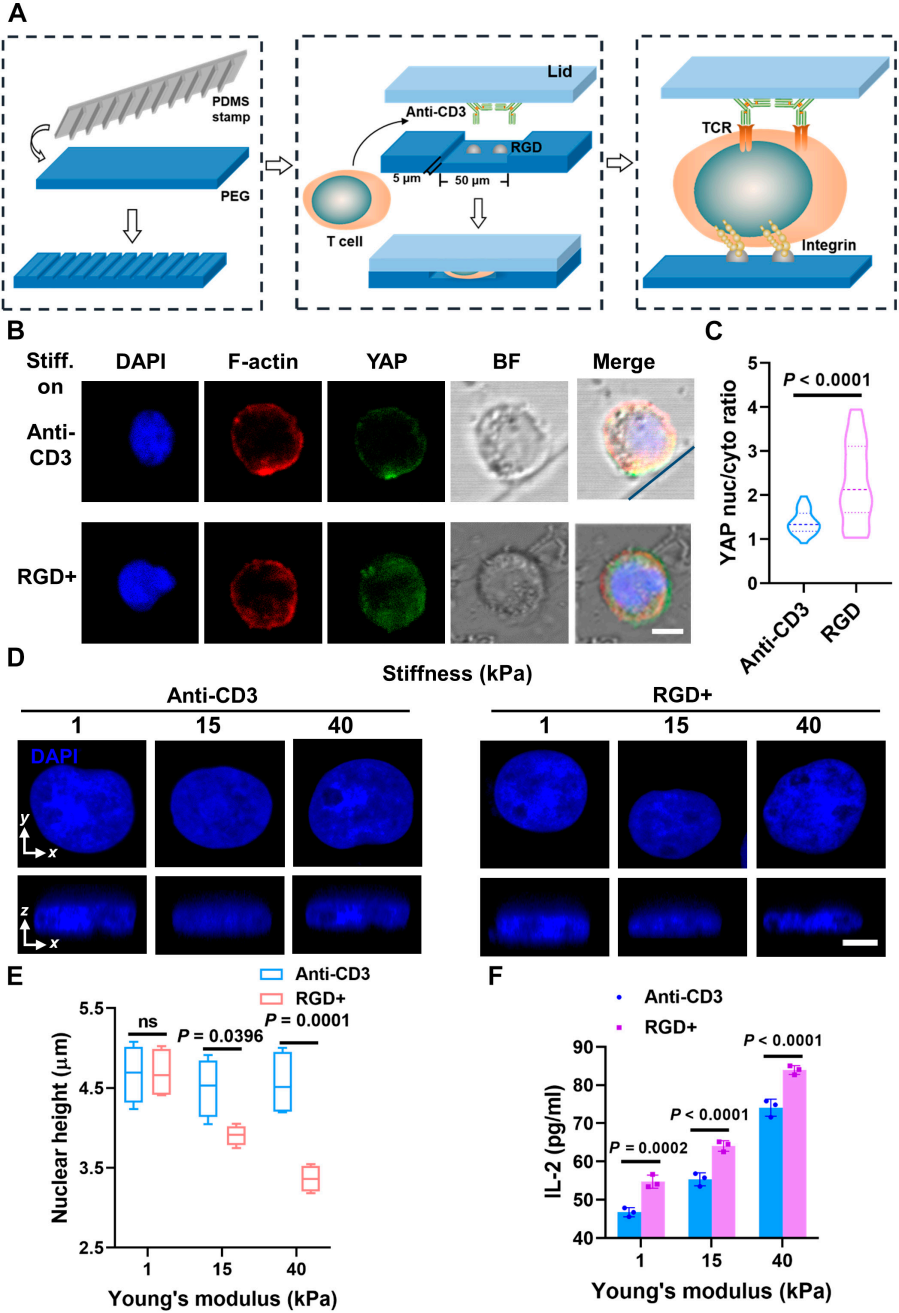
Construction of a microchannel hydrogel system for the study of matrix mechanotransduction on T cells. (A) Schematic of microchannel PEG hydrogel system fabrication. PDMS was used to obtain a PEG hydrogel with a microchannel structure by flipping the model twice, then the RGD peptide was modified at the bottom of the microchannel to mimic ECM, and anti-CD3 was modified at the lid of the microchannel to mimic APC. (B) Representative confocal images of T cells in the microchannel PEG hydrogel system, while “stiffness on anti-CD3” indicates that the lid of the microchannel modified with anti-CD3 had higher stiffness (40 kPa), while the bottom modified with RGD had lower stiffness (1 kPa). Conversely, “stiffness on RGD” indicates that the bottom of the microchannel modified with RGD had higher stiffness (40 kPa), and the lid modified with anti-CD3 had lower stiffness (1 kPa), as DAPI for nucleus (blue), F-actin (red), YAP (green), and BF (bright field). Blue lines were added to indicate the boundaries of the microfluidic channel to assist in spatial interpretation. (C) Corresponding quantification of YAP nuc/cyto for (B). (D) Representative DAPI staining at the cross-sectional side view was captured along the *xy* and *xz* plane crossing the center of the T cell’s nucleus in 1, 15, and 40 kPa of the lid or the bottom of the microchannel PEG hydrogels. (E) Corresponding quantification of nuclear height for (D). (F) Quantification of IL-2 expression of the T cells in 1, 15, and 40 kPa of the lid or the bottom of the microchannel PEG hydrogels. Anti-CD3 represents that anti-CD3 was modified at the lid with tunable stiffness of the microchannel. RGD+ indicates that RGD was modified at the bottom of the microchannel with tunable stiffness, which differed from the previous conditions. For the quantification of nuclear height and YAP nuc/cyto ratios, the number of T cells used for statistics was ≥30; these cells were selected from over 10 independent fluorescence microscopy images. Data are presented as means ± SEM, and *P* values were obtained by unpaired two-tailed Student’s *t* test (C) and using one-way ANOVA, followed by Tukey’s post hoc test (E and F). Scale bars, 10 μm (B) and 5 μm (D).

Our findings lead us to conclude that integrin-mediated matrix stiffness plays a pivotal role in shaping the mechanical microenvironment governing cell activation. Throughout the activation process, the dynamic mechanical forces are transmitted by T cell–ECM interactions, while the interactions between T cells and APCs persist (Fig. S10A). To provide direct evidence that it is integrins, rather than the TCR within the IS, that sense ECM stiffness during T cell activation, we constructed a microchannel PEG hydrogel system. This hydrogel system represents the interaction of T cells with both APC and the ECM of the LN microenvironment in vivo (Fig. 6A). By modifying the RGD peptide at the bottom of the microchannel to mimic the ECM and preparing another PEG hydrogel cap modified with anti-CD3 to mimic APCs, we engineered a system where T cell interactions with ECM and APCs occur simultaneously. This allowed us to regulate the stiffness of the ECM (at the bottom of the microchannel) independently of the IS interface, thereby mimicking the real physiological conditions more accurately and enabling further validation of our conclusions.

To ensure the proper activation of T cells within the microchannel system, we first characterized the structure of the microchannel and confirmed that it closely matches the original design, facilitating T cells’ access to both the bottom and the lid to replicate an in-vivo-like system (Fig. S10B). In addition, we successfully constructed microchannels of varying widths without altering their depth upon ligand modification (Fig. S10C and D). To verify our conclusion that matrix stiffness primarily regulates T cell activation through integrin-mediated T cell and ECM interaction, we separately modulated the lid and the bottom of the microchannel to achieve higher stiffness. Our results revealed that when high matrix stiffness was sensed by integrins, the YAP nucleoplasmic ratio was significantly higher compared to when high matrix stiffness was sensed by TCR (Fig. 6B and C). In Fig. 6B, we present both bright-field and fluorescence images of cells within the channel system. Our findings reveal that T cell activation is amplified through a synergy between IS signaling and integrin mechanotransduction. While initiation requires TCR-driven ZAP70 phosphorylation, ECM stiffness potentiates this response via a distinct integrin-mediated pathway. Stiffness triggers actin polymerization, nuclear deformation, and YAP translocation, independently of the synapse. This identifies integrin mechanosensing as a potent amplifier of T cell activation, elucidating how biochemical and mechanical cues are integrated for a full immune response (Fig. 6D to F).

While our microchannel platform decoupled integrin–ECM (RGD) and TCR–APC (anti-CD3) interactions to enable independent control of mechanical and biochemical cues, we acknowledge that this strict spatial separation does not fully recapitulate the in vivo microenvironment, where T cells integrate multiple concurrent signals. Nevertheless, this in vitro system allows precise modulation of substrate stiffness, providing mechanistic resolution that remains technically challenging to achieve in vivo. To partially address physiological relevance, we performed IHC and IF analyses on human LNs, which revealed stiffness-associated differences in T cell activation markers. The consistent activation patterns observed between the microchannel system, 2-dimensional (2D) substrates, and human LN tissues further strengthen the physiological significance of our results and highlight the mechanistic insights afforded by this approach. Besides, whether similar mechanosensing mechanisms occur in memory or immunosuppressed T cells, such as programmed cell death protein 1-positive (PD-1^+^) subsets, remains to be clarified. Future work with primary T cell populations will be needed to extend these findings to immunotherapy-relevant contexts. Together, our findings confirm that integrin-mediated matrix stiffness sensing induced nuclear deformation to promote T cell activation via F-actin aggregation, highlighting the important role of integrin-mediated mechanotransduction in regulating T cell activation and suggesting future in vivo strategies to directly modulate nuclear deformation for potential clinical applications.

## Conclusion

This study presents a novel revolution of the mechanotransduction governing integrin-mediated matrix-stiffness-driven T cell activation, offering insights into a fundamental aspect of immunology. While T cell activation traditionally involves TCR engagement with p-MHC ligands presented by APCs, our findings highlight the crucial role of ECM stiffness variations, particularly in the context of metastases within LNs. We demonstrate that T cells sense changes in LN matrix stiffness through RGD/integrin binding, triggering a cascade of events. Elevated matrix stiffness enhances F-actin aggregation, which deforms the nucleus and thereby promotes the translocation of YAP into the nucleus. This, in turn, drives IL-2 expression, resulting in T cell activation (Fig. S11).

Our study not only advances the fundamentals of T cell activation but also provides a theoretical framework for comprehending how the mechanical microenvironment regulates the phenotype of immune cells. Furthermore, by elucidating the key role of YAP-mediated signaling and nucleus deformation in T cell activation, our findings provide potential avenues for modulating T cell activation through biomechanical regulation. Such mechanistic understanding may inform the future development of engineered culture platforms or immunotherapeutic strategies that leverage controlled mechanical inputs, while in vivo validation and functional efficacy studies remain important directions for future investigation.

## Methods

### Assessment of LN metastasis and treatment response

LN metastasis status and treatment efficacy were evaluated in all patients with lung cancer receiving first-line anti-PD-1 inhibitor combined with chemotherapy. Each treatment cycle lasted 21 d, and tumor responses were assessed every 2 cycles. Baseline-contrast-enhanced CT or positron emission tomography–CT images before treatment were independently reviewed by 2 experienced radiologists. LN metastasis was defined as LNs with a short-axis diameter of ≥1 cm. A total of 45 patients with tumor-metastasis-positive LN and 18 patients with tumor-metastasis-negative LN were included in the analysis.

### Collection and characterization of the LN samples

We collected all the LN samples from 6 patients who had undergone radical resection of lung cancer as initial treatment in the First Affiliated Hospital of Xi’an Jiaotong University, which the Research Ethics Committee of the First Affiliated Hospital of Xi’an Jiaotong University approved (LLSBPJ-2023-496). We divided each obtained LN into 2 pieces. One piece was fixed and embedded with paraffin. Then, the hematoxylin and eosin staining was performed to confirm the status of tumor metastasis in LNs, and the primary antibodies CD8 (Thermo Fisher Scientific), CD69 (Proteintech), and CD38 (Proteintech) were used in IHC staining or IF following standard protocols to characterize the level of T cell activation according to the manufacturer’s instructions. Briefly, formalin-fixed paraffin-embedded LN sections were deparaffinized, rehydrated, subjected to antigen retrieval, and incubated with primary antibodies, followed by appropriate secondary antibodies and chromogenic detection. For quantitative analysis, IHC and IF images were analyzed in anatomically defined regions of interest (ROIs) of each LN section. ROIs were manually delineated using ImageJ (National Institutes of Health), and mean fluorescence intensity or IHC signal density was measured for each region. For each sample, multiple fields of view from at least 3 independent sections were analyzed to ensure statistical robustness.

### Preparation of peptide and antibody-modified PEG hydrogel

For preparation of PEG hydrogels, 8-arm PEG-MAL (10 kDa; Pengsheng Biological) and 8-arm PEG-SH (10 kDa; Pengsheng Biological) at the concentration of 10 wt % were mixed for 30 min at room temperature via Michael addition. The hydrogels were rinsed thrice with phosphate-buffered saline (PBS) following each step to remove unreacted molecules. It is necessary to modify the CD3 monoclonal antibody on the surface of PEG hydrogels to provide sustained TCR engagement and initiate T cell activation, whereas soluble anti-CD28 is widely used as a uniform costimulatory signal and therefore was added to the culture medium at a final concentration of 2 μl/ml rather than immobilized on the hydrogel. To avoid competitive binding effects and ensure consistent anti-CD3 density, we carefully optimized the cofunctionalization process. Specifically, 10 mM cRGD peptides (GCGYGRGDSSPG) (Sangon Biotech, Shanghai) were first modified to the hydrogel surface via addition reaction overnight to establish integrin adhesions. Subsequently, biotinylated PEG-SH (biotin-PEG-SH; JenKem Technology), streptavidin (Yeasen Biotechnology), and CD3 monoclonal antibody (Thermo Fisher Scientific) were added into PBS buffer at a final concentration of 0.15, 0.3, and 0.15 nM, respectively, to achieve stable and reproducible immobilization of anti-CD3 on the hydrogel surface. Using the biotin–affinity–biotin reaction system, PEG hydrogels were soaked in these buffers step by step. To validate that the presence of RGD peptides did not interfere with the anti-CD3 coating efficiency, rhodamine-labeled RGD and fluorescein isothiocyanate (FITC)-labeled CD3 monoclonal antibody were used to characterize the conjugation of RGD or CD3 monoclonal antibody in the hydrogels. For the experiments involving ligand functionalization (anti-CD3 and RGD peptides), the stiffness of the hydrogels was fixed at 15 kPa, unless otherwise specified.

### Mechanical properties of hydrogels

The stiffness of LN tissue was measured at the microscale using the Nanoindenter (Optics11, Netherlands). Indentation experiments were performed randomly at the subregion of LNs. Briefly, LNs were first embedded in optimal cutting temperature (OCT) compound and then sectioned into 100-μm-thick slices using a cryostat. Before measurement, tissue sections were firmly adhered to plastic dishes to prevent displacement during indentation. A nanoindenter equipped with a spherical probe tip (radius *R*) was used to randomly select multiple points within the LN tissue region for indentation measurements. The force–displacement curves obtained were converted into force–indentation depth curves and fitted using the Hertz model. The Young’s modulus (*E*) of the tissue was calculated according to the following equation:

P=43E1−ν2R12h32 (1)

where *P* is the load on the LN, *R* is the radius of the spherical tip, *h* is the indentation depth, and *E* and *υ* are the Young’s modulus and Poisson’s ratio (here, its value is set to 0.5) of the LN.

Besides, a rotational rheometer (Anton Paar MCR 302, Austria) was used for rheological testing at a temperature of ~25 °C using 8-mm stainless steel parallel plates. The storage modulus *G*′ and loss modulus *G″* were obtained by measurement at 1% strain and a frequency of 1 rad/s. The following formula was used to calculate the corresponding stiffnesses:

$$G=\sqrt{G^{\prime 2}+G^{\prime \prime 2}}$$ (2)

$$E=2G\left(1+\nu \right)$$ (3)

where *E* is the elastic modulus of PEG hydrogel to be determined, ν represents the material’s Poisson ratio (here, its value is set to 0.5), and *G* is the modulus of PEG hydrogel.

### T cell culture

The T cells (Jurkat T cells, clone E6-1) are provided by the National Collection of Authenticated Cell Cultures (Shanghai). T cells were cultured in the cell flasks using RPMI 1640 complete medium, which contains 1% sodium pyruvate (100 mM), 1% penicillin (10 kU/ml)/streptomycin (10 mg/ml), and 5% fetal bovine serum (Gibco, USA) at the condition of 37 °C and 5% CO_2_. Because of the density-dependent nature of these T cells, live cells were controlled to grow at a density of 1 × 10^6^/ml and passaged using a half-exchange method. When the density of cell growth is appropriate and the number of live cells is above 90%, T cells were seeded on PEG hydrogels with different conditions for further experiments. Unless otherwise stated, all experiments were performed using Jurkat T cells. In experiments where primary murine T cells were used, this has been separately indicated.

### IF staining and enzyme-linked immunosorbent assay

For IF staining of T cells, cells seeded on functional PEG were first fixed with 4% paraformaldehyde (BIOSHAP) for 10 min, followed by 15-min permeabilization with 0.5% Triton X-100 (Sigma-Aldrich). Then, the samples were blocked with 5% bovine serum albumin (BSA; MP Biomedicals) for 50 min at 37 °C. The primary antibodies used in our study include p-Zap70 antibody (1:50; 2701/2717, Cell Signaling Technology, USA), Zap70 antibody (1:200; 3165, Cell Signaling Technology, USA), YAP antibody (1:100; 14074, Cell Signaling Technology, USA), and anti-IL-2 receptor α antibody (CD25; 1:50; ab231441, Abcam, USA) at 4 °C overnight. Alexa Fluor 488 (H + L) secondary antibody (1:500; A11034, Cell Signaling Technology, USA) was further incubated at room temperature for 2 h. Cell cytoskeleton was stained by rhodamine phalloidin (1:500; R415, Invitrogen, USA). 4′,6-Diamidino-2-phenylindole (DAPI; 0.5 μg/ml; 4083, Cell Signaling Technology, USA) was used to stain cell nuclei. For enzyme-linked immunosorbent assay (ELISA), Supernatant was harvested by centrifugation at 4 °C and 12,000*g* for 10 min, and then the expression level of IL-2 was analyzed by a commercial ELISA kit (YJ 88-7025-88, Thermo Fisher Scientific) according to the manufacturer’s instructions.

All fluorescence imaging was performed using an Olympus FV3000 confocal laser scanning microscope (Olympus, Japan). A 60× oil immersion objective lens was utilized for high-resolution imaging. Fluorescence excitation was achieved using laser lines at 405 nm (50 mW), 488 nm (20 mW), and 561 nm (20 mW), with laser power settings optimized for each fluorophore. Emission was collected using appropriate bandpass filters corresponding to each fluorophore. To ensure consistency, all cells and hydrogels were imaged under identical settings, including laser intensity, detector gain, and exposure time. Z-stack images were acquired with a step size of 0.5 μm to capture the entire cell nucleus. Unless otherwise specified, images presented are maximum intensity projections of the z-stacks. All images include scale bars for reference. For fluorescence quantification, the mean fluorescence intensity of individual T cells was quantified from raw confocal images using ImageJ. ROIs were manually delineated around each cell to obtain the mean intensity values for markers of interest (e.g., p-Zap70, F-actin, YAP, and CD25). A minimum of 30 cells from more than 10 randomly selected images per condition were analyzed to ensure statistical robustness.

### Quantification of nuclear/cytoplasmic YAP ratio

Immunofluorescent staining for YAP/F-actin/DAPI was used to calculate the ratio of nuclear YAP to cytoplasmic YAP (YAP nuc/cyto). Then, these areas were determined by using ImageJ. The following formula was used to calculate the YAP nuc/cyto ratio:

$$\text{Ratio of}\;\mathrm{YAP}\;\mathrm{nuc}/\text{cyto}=\frac{\Sigma_{\mathrm{nuc}}^{\prime }/A_{\mathrm{nuc}}}{\left(\Sigma_{\mathrm{tot}}^{\prime }-\Sigma_{\mathrm{nuc}}^{\prime }\right)/\left(A_{\mathrm{tot}}-A_{\mathrm{nuc}}\right)}$$ (4)

where $\Sigma_{\mathrm{nuc}}^{\prime }$ is the total YAP fluorescence intensity of all pixels within the nucleus, as delineated by DAPI staining; $\Sigma_{\mathrm{tot}}^{\prime }$ was that for the entire cell, delineated by F-actin staining; *A*_nuc_ was the number of pixels in the DAPI stained nucleus; and *A*_tot_ was the number of pixels in the entire F-actin stained cell. The number of T cells used for statistics was ≥30, and these cells were selected from over 10 independent fluorescence microscopy images.

### Construction and characterization of hydrogel microchannel

Our study aims to elucidate the distinct mechanical contributions of integrin-mediated adhesion signaling in T cell activation. To achieve this, we used a 2-pronged approach. We initially investigated the role of RGD-mediated integrin adhesion on T cell activation using 2D substrates. This setup allowed us to observe how integrin engagement influences T cell behavior in a controlled environment. To further dissect the mechanical interplay between integrin and TCR signaling, we designed microchannels that mimic the confined spaces T cells navigate in vivo. This setup enabled us to compare the mechanical forces mediated by integrins and TCRs during T cell activation. Polydimethylsiloxane (PDMS; DC-184, Dow Corning) stamp (cross-linking agent/monomer: 1/10) was cast using silicon master, which was featured with grooves (width: 10, 30, 50, 100 and 500 μm; depth: 5 μm) by photoetching. Then, the PDMS stamp was coated with 2% BSA (MP Biomedicals) for 60 min at 37 °C. Afterward, the PEG hydrogels mixed by the same method were micromolded into a grooved substrate with defined dimensions by the PDMS stamp overnight. Finally, a flat PEG hydrogel lid was placed on the top of the hydrogel grooves to form the 3D microchannels. Rhodamine-labeled RGD was modified on PEG hydrogel microchannels to characterize the dimensions by a laser scanning confocal microscopy (FV3000, Olympus) with 1-μm z-stacks. Images of microchannels were 3D reconstructed and analyzed by cellSens software (v. 1.18, Olympus).

### Simulation of nuclear deformation

To simulate the experimental observations of T cell nucleus deformation driven by actin polymerization during activation, we developed a qualitative model using COMSOL Multiphysics v.5.4 structural mechanics module. In this model, actin forces were partitioned into *P*, *Q*, and *T* loads, as depicted in Fig. 5A. The cell nucleus was modeled as a 2D ring structure with mesh division, and the displacement field induced by the applied loads was computed to understand the mechanical impact on the nucleus. This modeling approach aligns with previous studies that have utilized finite element analysis to understand nuclear deformation in T cells.

We detailed this modeling approach in Note S1 and Table S1. This comprehensive methodology provides insights into the mechanical aspects of T cell activation and the distinct roles of integrin and TCR-mediated forces.

### Statistical analysis

The GraphPad Prism 8 (GraphPad Software, San Diego, CA, USA) or Microsoft Excel 2019 was used for statistical analyses. Data were shown as the means ± SEM, unless otherwise stated. Prior to selecting the appropriate statistical tests, we assessed the normality of the data distributions using the Shapiro–Wilk test. For datasets that followed a normal distribution, we applied parametric tests, such as the unpaired 2-tailed Student’s *t* test for comparisons between 2 independent groups and one-way analysis of variance (ANOVA) with Tukey’s post hoc test for multiple group comparisons. For datasets that did not meet the assumption of normality, we used nonparametric tests, including the Mann–Whitney *U* test for independent samples and the Wilcoxon signed-rank test for paired samples. The threshold for statistically significant differences between groups was *P <* 0.05. All experiments were repeated independently at least thrice, and the number of cells counted for each condition was indicated in each figure legend.

## Data Availability

All data needed to evaluate the conclusions in the paper are present in the paper or the Supplementary Materials.
