# Supplementary material for: Integrin-Mediated Mechanosensing of Modeled Lymph Node Microenvironment Promotes T Cell Activation via Nuclear Deformation
Source: Research (Wash D C). 2026 Feb 6;9:1121. doi: 10.34133/research.1121 (PMC12877341; doi:10.34133/research.1121)
Supplement: Supplementary 1 — Figs. S1 to S11 Supplementary Text Table S1 [file research.1121.f1.zip › Revised Manuscript-SM (Clean Version).docx]

Supplementary Materials for

**Integrin-mediate Mechanosensing of Modeled Lymph Node Microenvironment Promotes T-Cell Activation via Nuclear Deformation**

Jinteng Feng *et al.*

*Corresponding author. Hui Guo, Email: guohui@xjtufh.edu.cn; Min Lin, Email: minlin@xjtu.edu.cn.

**This PDF file includes:**

Figs. S1 to S11

Supplementary Text

Tables S1

**Supplementary Figures**

Fig. S1.


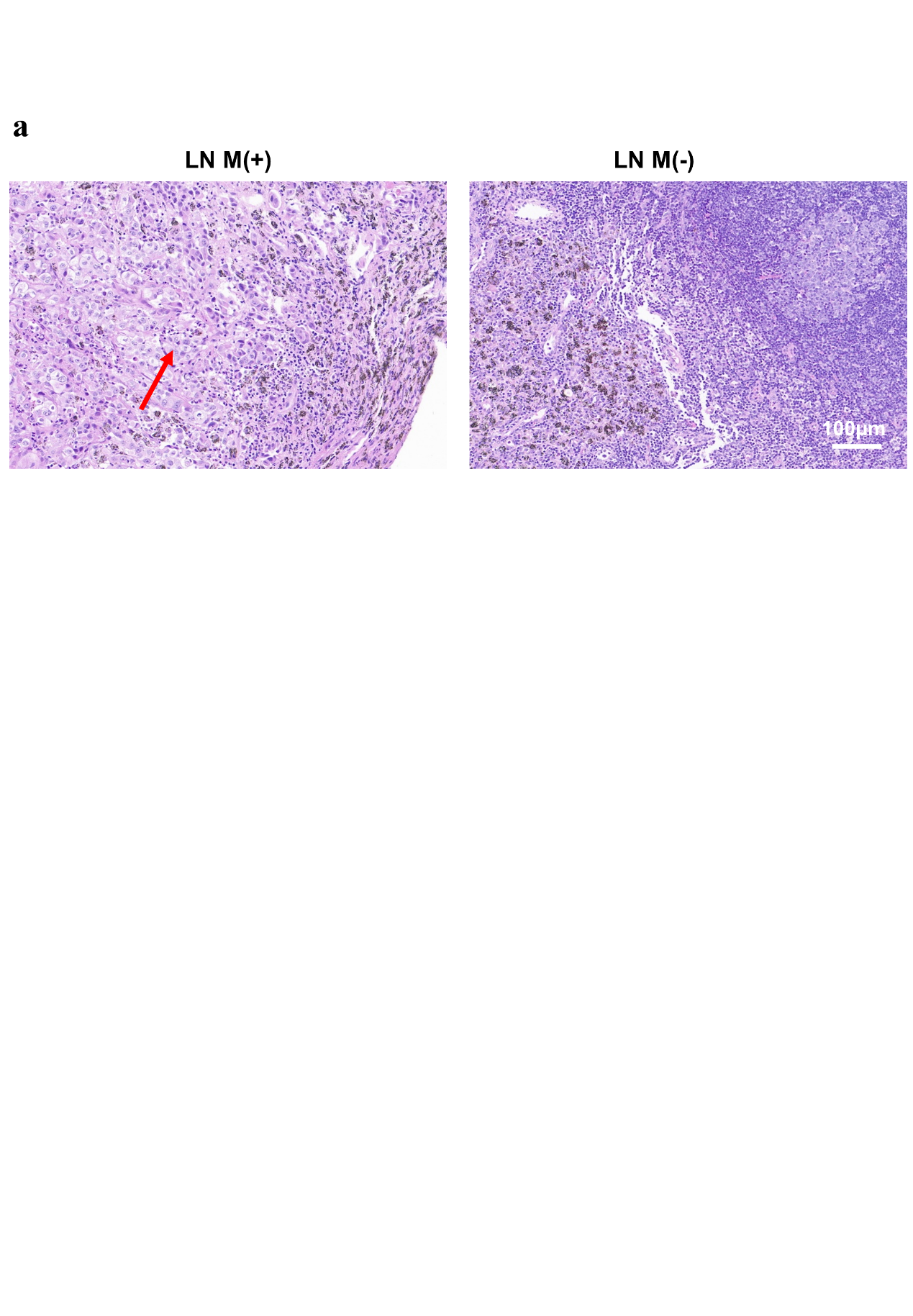


**Supplementary Fig. 1 | a**. Representative HE staining lymph nodes. The white arrow indicates metastatic tumor. LNM(+) represents tumor metastasis-positive lymph node. LNM(-) represents tumor metastasis-negative lymph node. Scale bars showed on the images.

Fig. S2.


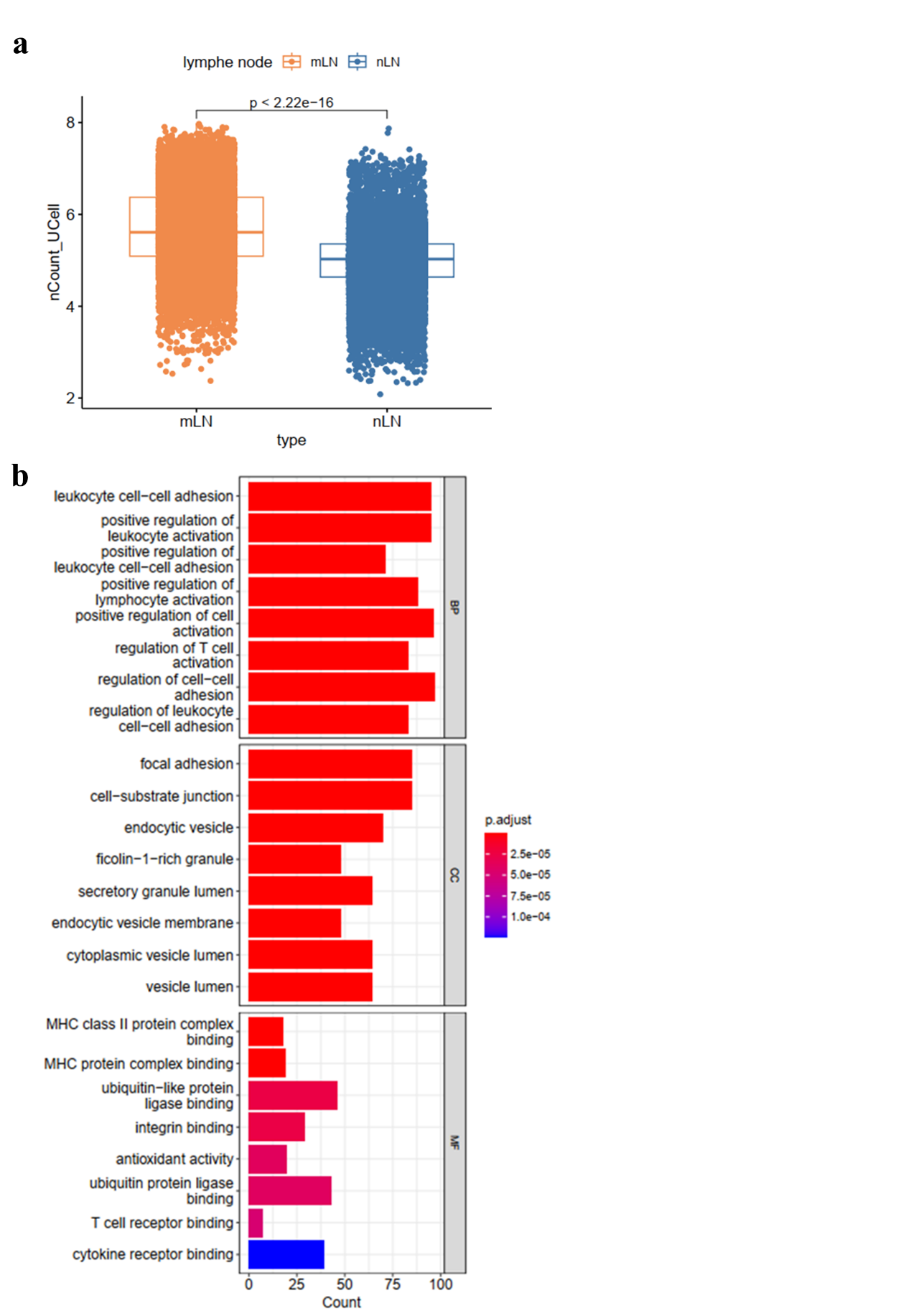


**Supplementary Fig. 2 |** **a**. Comparison of stiffness-associated transcriptional scores between metastatic lymph nodes (mLN) and normal lymph nodes (nLN) using single-cell RNA-seq data from the GSE131907 dataset. Data are presented as mean ±  S.E.M., and *P*-values were obtained by Two-tailed Student’s *t*-test **b**. GO enrichment analysis between groups of different stiffness-related scores.

Fig. S3.


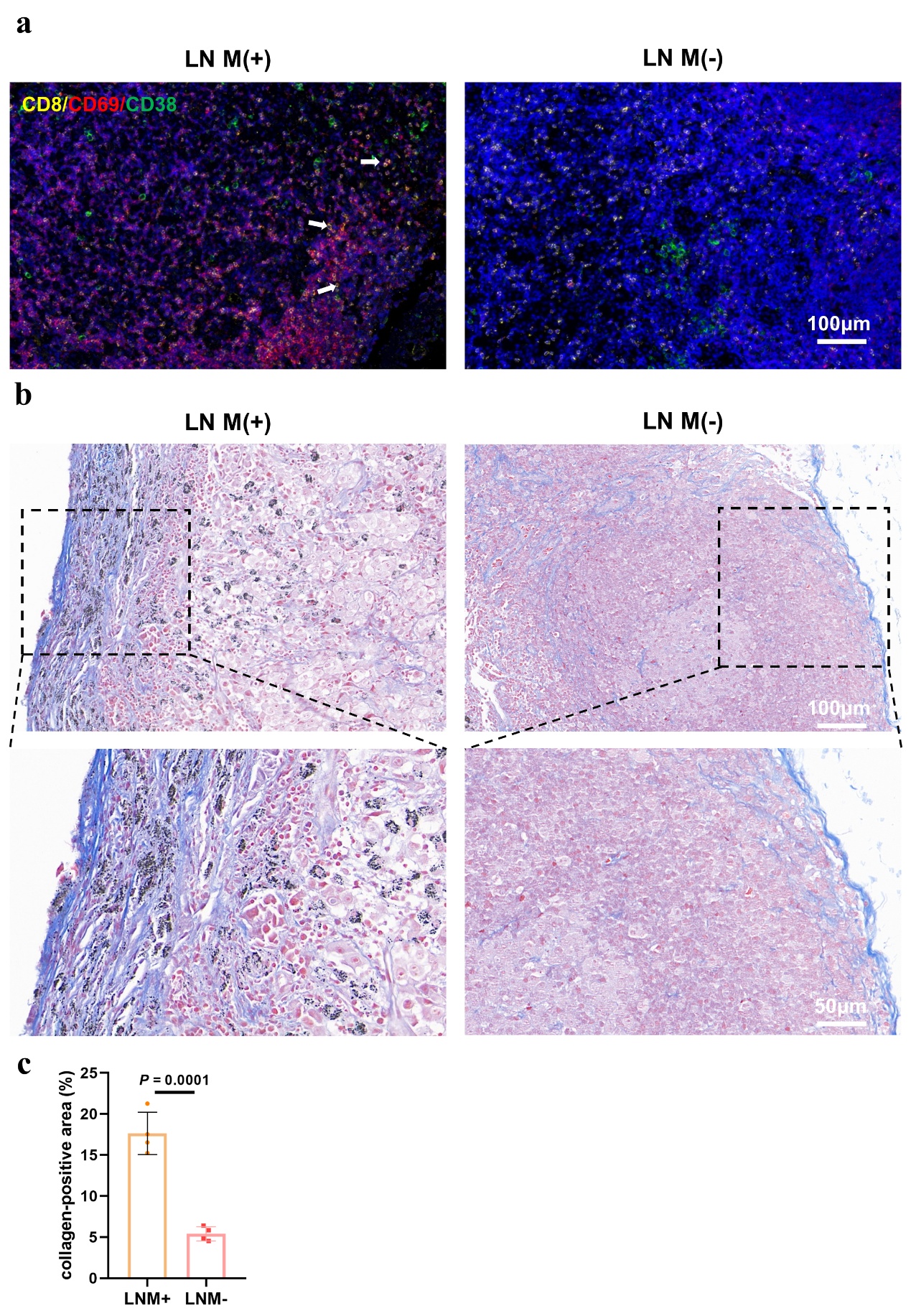


**Supplementary Fig. 3 | a.** Representative multiplex immunofluorescence staining of lymph nodes, as CD8 (yellow), CD69 (red), CD38 (green), and DAPI for nucleus (blue). White arrows indicate double-positive cells. **b.** Representative Masson’s staining images in lymph nodes from LNM(+) and LNM(-) groups. **c.** Quantification of collagen-positive area (%) in lymph nodes from LNM(+) and LNM(-) groups. Collagen (blue) areas were quantified using ImageJ. Data are presented as mean ± SEM. Scale bars showed on the images.

Fig. S4.


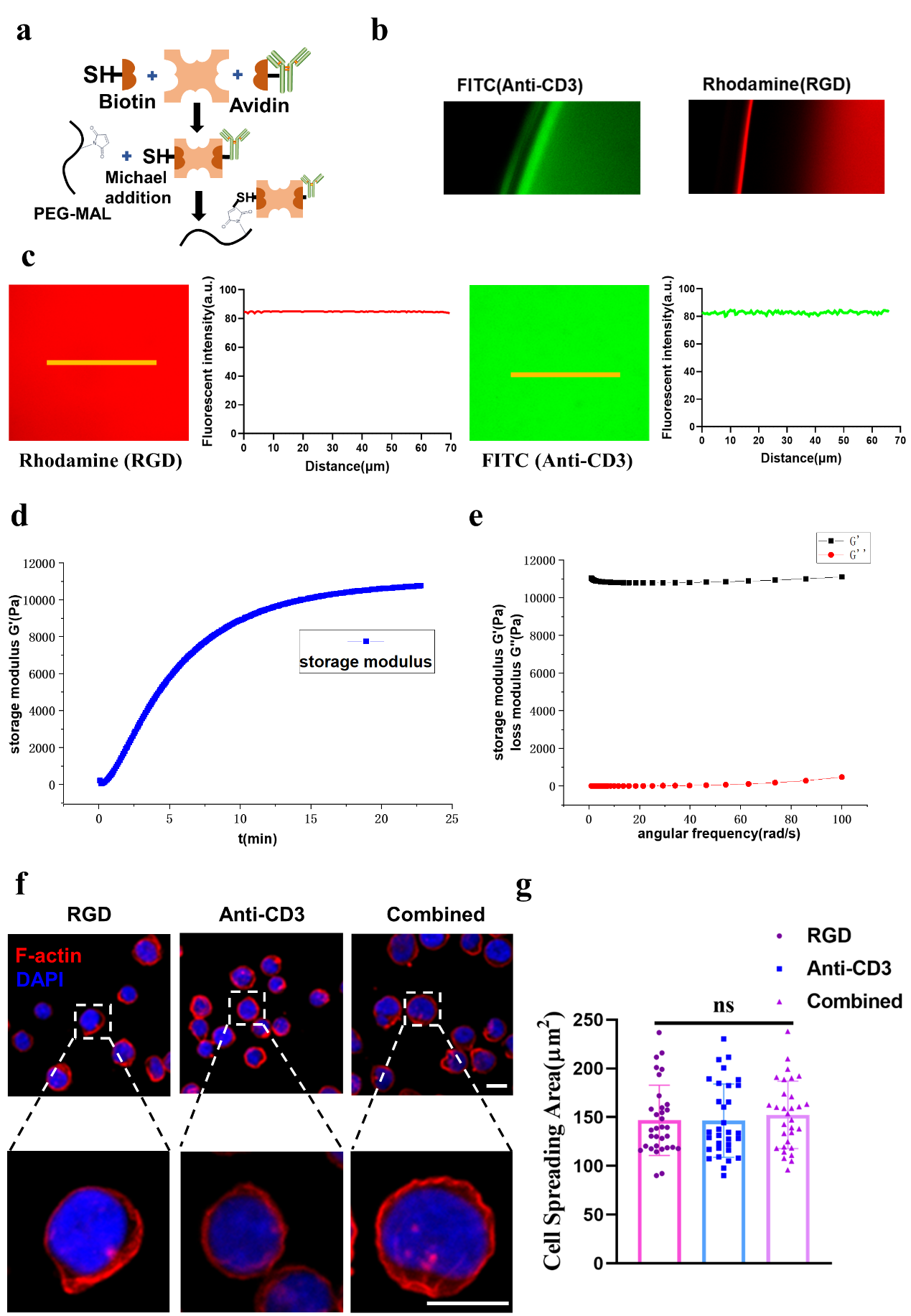


**Supplementary Fig. 4 | a.** Schematic of modifying the anti-CD3 monoclonal antibody in PEG hydrogels using biotin-affinity-biotin system via Michael addition reaction. **b.** Representative fluorescence images of the boundaries of PEG hydrogels for visualization of RGD and anti-CD3. The anti-CD3 monoclonal antibody was coupled with FITC (green fluorescence) and the RGD peptide was coupled with rhodamine (red fluorescence). **c.** Fluorescence images and intensity profiles of RGD and Anti-CD3 conjugation on PEG hydrogel surfaces. Left images show representative fluorescence images of rhodamine-labeled RGD (red) and FITC-labeled Anti-CD3 (green), conjugated separately onto flat PEG hydrogel surfaces. The yellow line indicates the region along which fluorescence intensity was measured. The right diagrams display the corresponding fluorescence intensity profiles across the yellow lines. **d.** The variation of storage modulus during PEG hydrogel cross-linking. **e.** Characterization of the storage modulus and loss modulus for the PEG hydrogel using rotational rheometer by measurement at 1% strain and a frequency of 1 rad s−1. **f.** Representative confocal images of T cells spreading on different modified substrates, such as DAPI for nucleus (blue) and F-actin (red). (RGD) represents only RGD peptide were modified on the substrates, (Anti-CD3) represents only anti-CD3 monoclonal antibody were modified on the substrates, and (Combined) represents both RGD peptide and anti-CD3 monoclonal antibody were modified on the substrates. **g.** Corresponding quantification of T cells spreading area for Fig. **f.** For the quantification of cell spreading area, the number of T cells used for statistics was ≥ 30. Cell spreading areas were measured 60 minutes post-seeding. Data are presented as mean ±  S.E.M., and *P*-values were obtained by Two-tailed Student’s *t*-test (g). Scale bars: 10 µm.

Fig. S5.


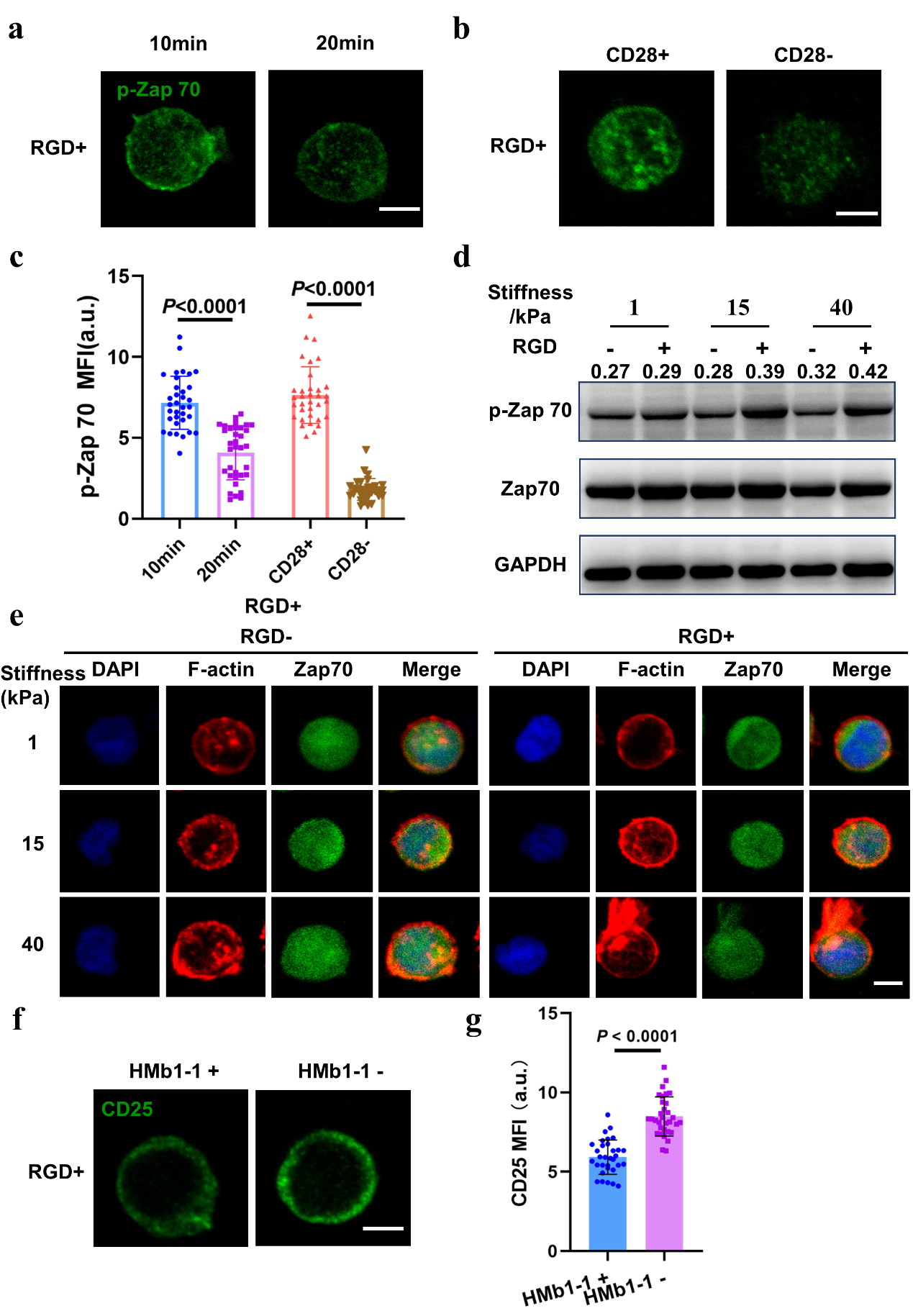


**Supplementary Fig. 5 | a.** Representative p-Zap 70 images after T cell activated for 10 mins or 20 mins on the（RGD+）substrates. **b.** Representative p-Zap 70 images of T cells treated with soluble CD28 monoclonal antibody or not on the（RGD+）substrates. **c.** Corresponding quantification of p-Zap 70 for Fig. a and Fig. b. **d.** The protein level of p-Zap 70 of T cells on 1, 15, and 40 kPa of different modified substrates. **e.** Representative confocal images of T cells on 1, 15, and 40 kPa of different modified substrates, as DAPI for nucleus (blue), F-actin (red), and Zap 70 (green). **f.** Representative CD25 (green) images of T cells, which were treated with β1 integrin–neutralizing antibody (HMb1-1). **g.** Corresponding quantification of CD25 70 for Fig. f. (RGD-) represents only anti-CD3 monoclonal antibody was modified on the substrates, and (RGD+) represents both RGD peptide and anti-CD3 monoclonal antibody were modified on the substrates. For the quantification of p-Zap 70 and CD25, the number of T cells used for statistics was ≥ 30. Data are presented as mean ±  S.E.M., and P-values were obtained by Two-tailed Student’s *t*-test (c). Scale bars: 10 µm.

Fig. S6.


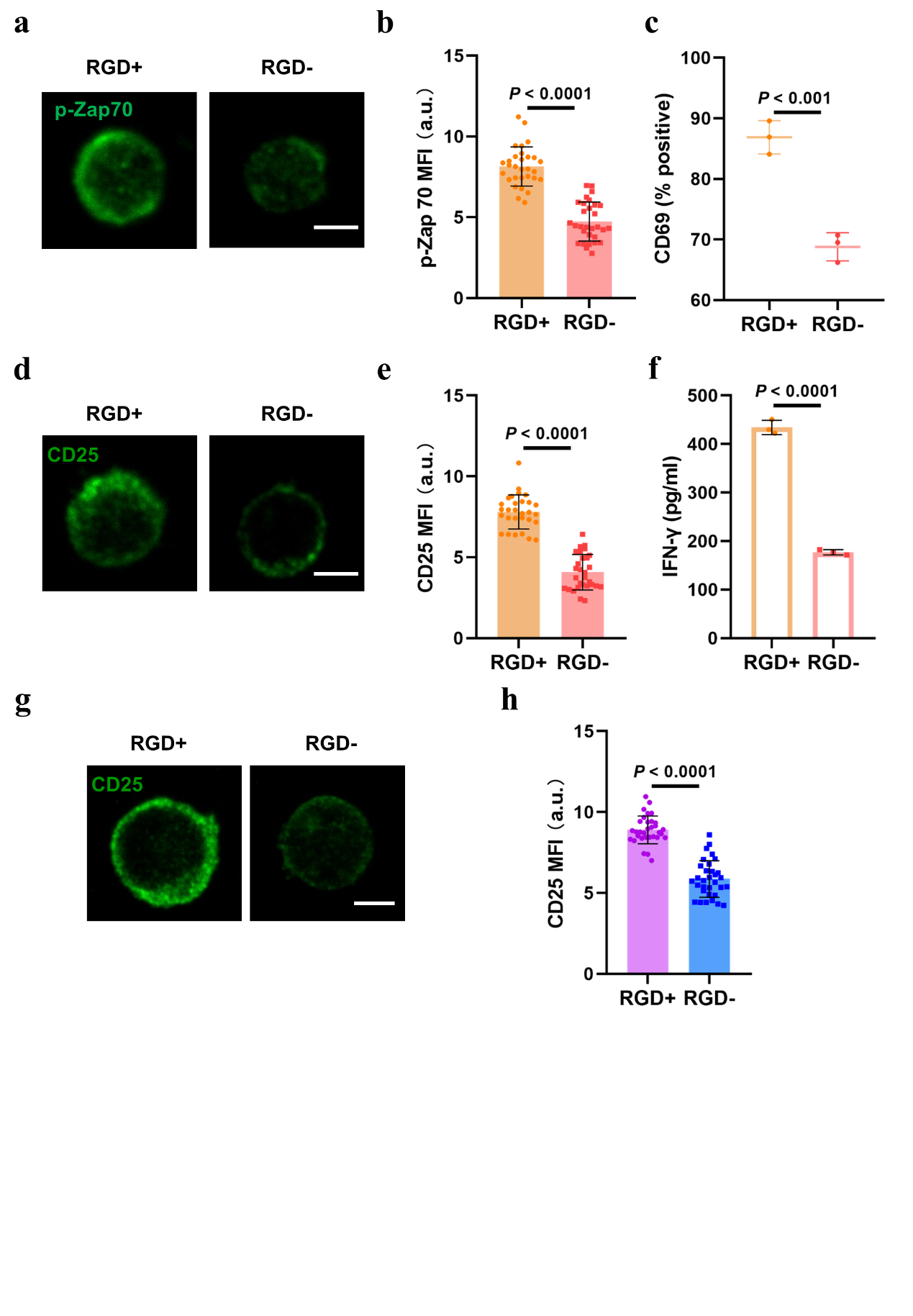


**Supplementary Fig. 6 | a.** Representative p-Zap 70 images of primary murine CD8^+^ T cells. **b.** Corresponding quantification of p-Zap 70 for Fig. a. **c.** Quantification of CD69 expression of primary murine CD8^+^ T cells. **d.** Representative CD25 images of primary murine CD8^+^ T cells. **e.** Corresponding quantification of CD25 for Fig. d. **f.** Quantification of IFN-γ expression of primary murine CD8^+^ T cells. **g.** Representative CD25 images of Jurkat T cells. **h.** Corresponding quantification of CD25 for Fig. g. (RGD-) represents only anti-CD3 monoclonal antibody modified on the substrates, and (RGD+) represents both the RGD peptide and the anti-CD3 monoclonal antibody modified on the substrates. All images were acquired and processed under identical conditions. For the quantification of p-Zap 70 and CD25, the number of T cells used for statistics was ≥ 30; these cells were selected from over 10 independent fluorescence microscopy images. Data are presented as mean ±  S.E.M., and *P*-values were obtained by Two-tailed Student’s *t*-test (b, c, e, f, h). Scale bars: 5 µm in Fig. a and Fig. d, and 10 µm in Fig. g.

Fig. S7.


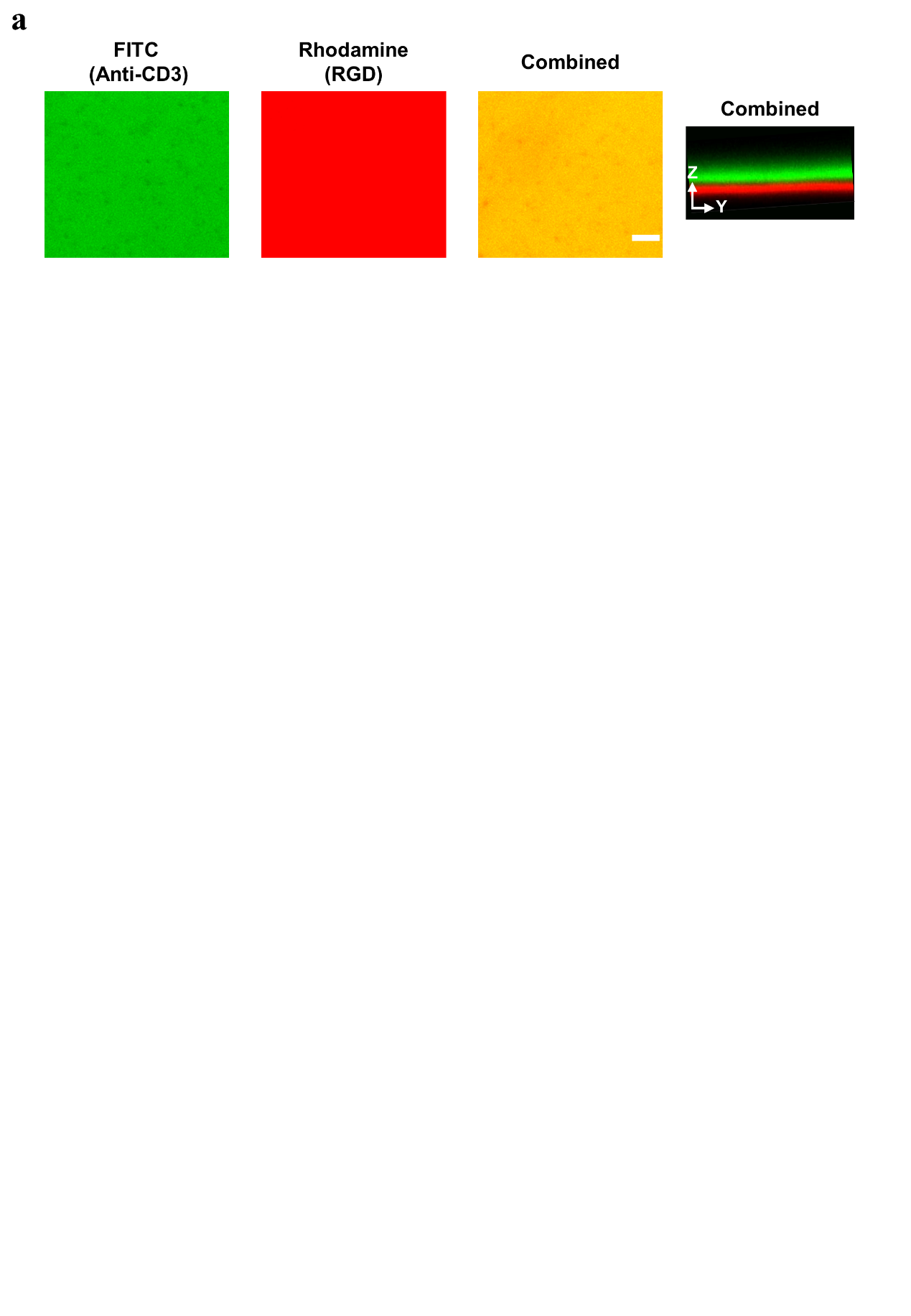


**Supplementary Fig. 7 | a.** Fluorescence images to characterize the distance between two layers of PEG hydrogel in the Z-axis. The anti-CD3 monoclonal antibody coupled with FITC was modified in the upper hydrogel (green fluorescence), the RGD peptide coupled with rhodamine was modified in the lower hydrogel (red fluorescence), and the distance was showed by image reconstruction with laser scanning confocal microscopy. Scale bar: 50 µm.

Fig. S8.


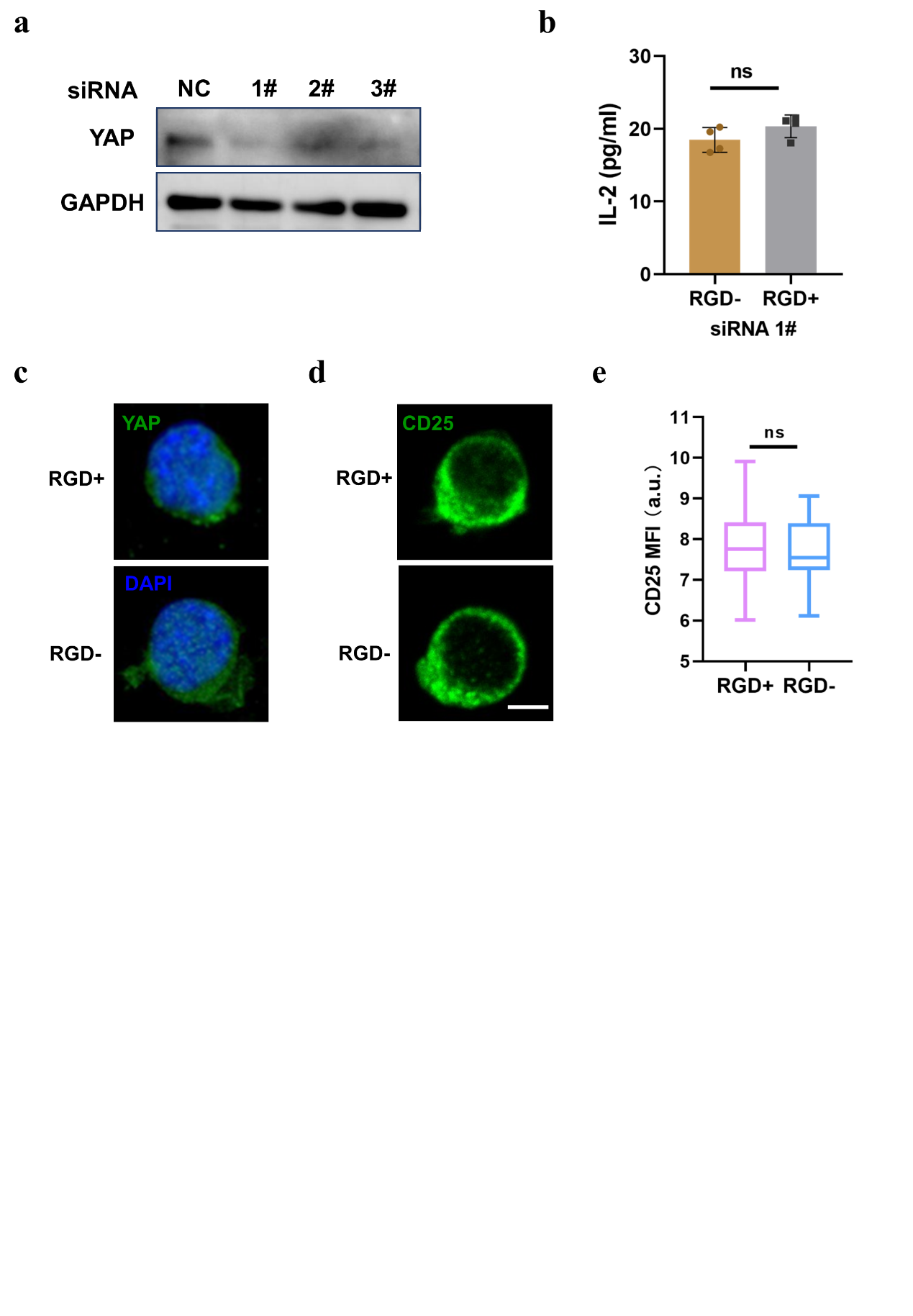


**Supplementary Fig.** **8 | a.** Representative Western Blotting image showed the protein level of YAP of T cells, which were treated with small interfering RNA (siRNA) to inhibit YAP gene expression. **b**. Quantification of IL-2 expression of the T cells treated with the exact siRNA (siRNA 1#) that could knock down the expression of YAP, apparently. **c.** and **d.** Representative immunofluorescence images of YAP (green) and CD25 (red) in T cells treated with Leptomycin B (LMB). **e.** Corresponding quantitative analysis of CD25 for Fig. d. (RGD-) represents only anti-CD3 monoclonal antibody modified on the substrates, and (RGD+) represents both RGD peptide and anti-CD3 monoclonal antibody modified on the substrates. DAPI for nucleus (blue). The number of T cells used for statistics was ≥ 30, these cells were selected from over 10 independent fluorescence microscopy images. Data are presented as mean ±  S.E.M., and *P*-values were obtained by Two-tailed Student’s *t*-test (b, e). Scale bars: 10 µm in Fig. c, and Fig. d.

Fig. S9.


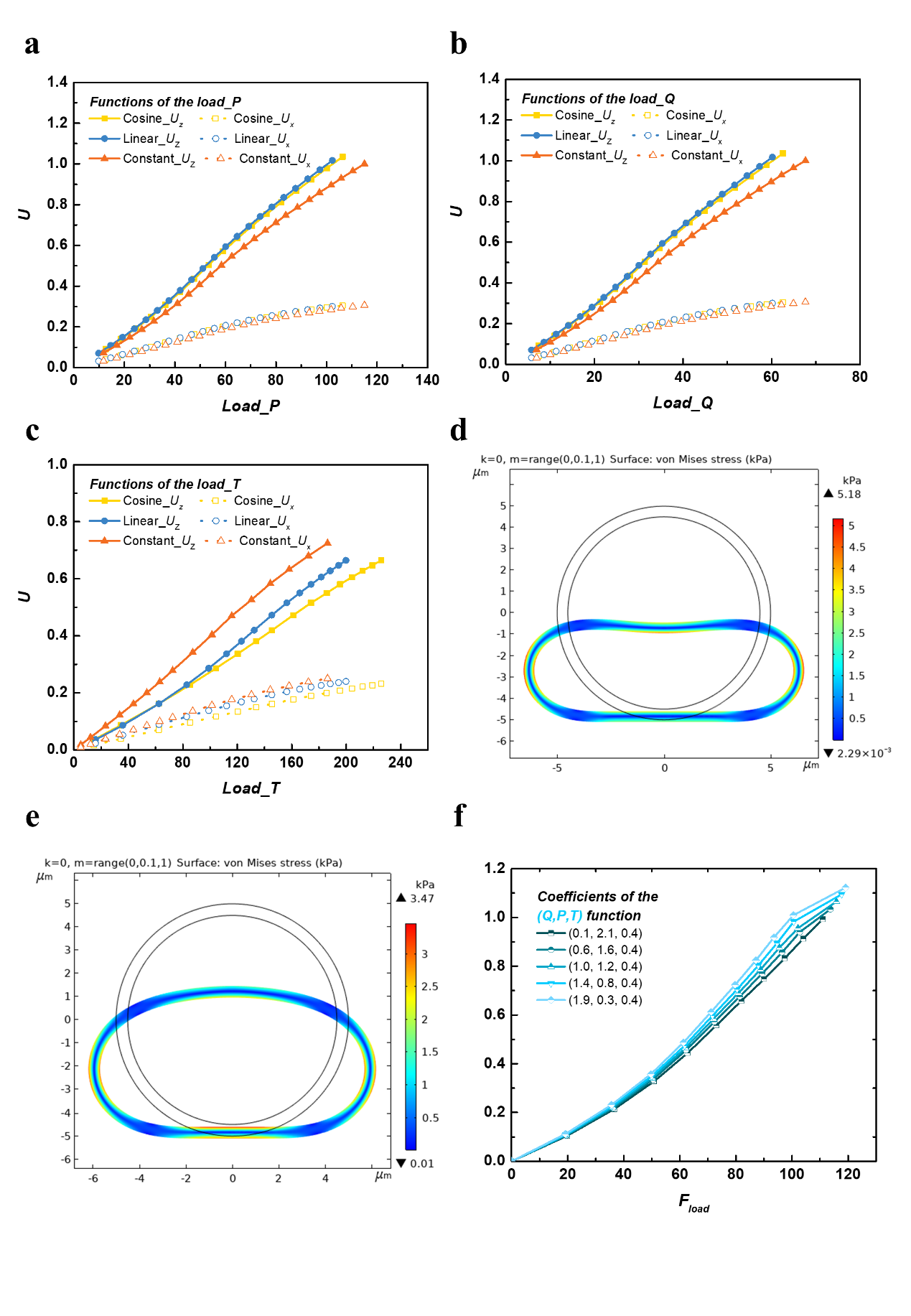


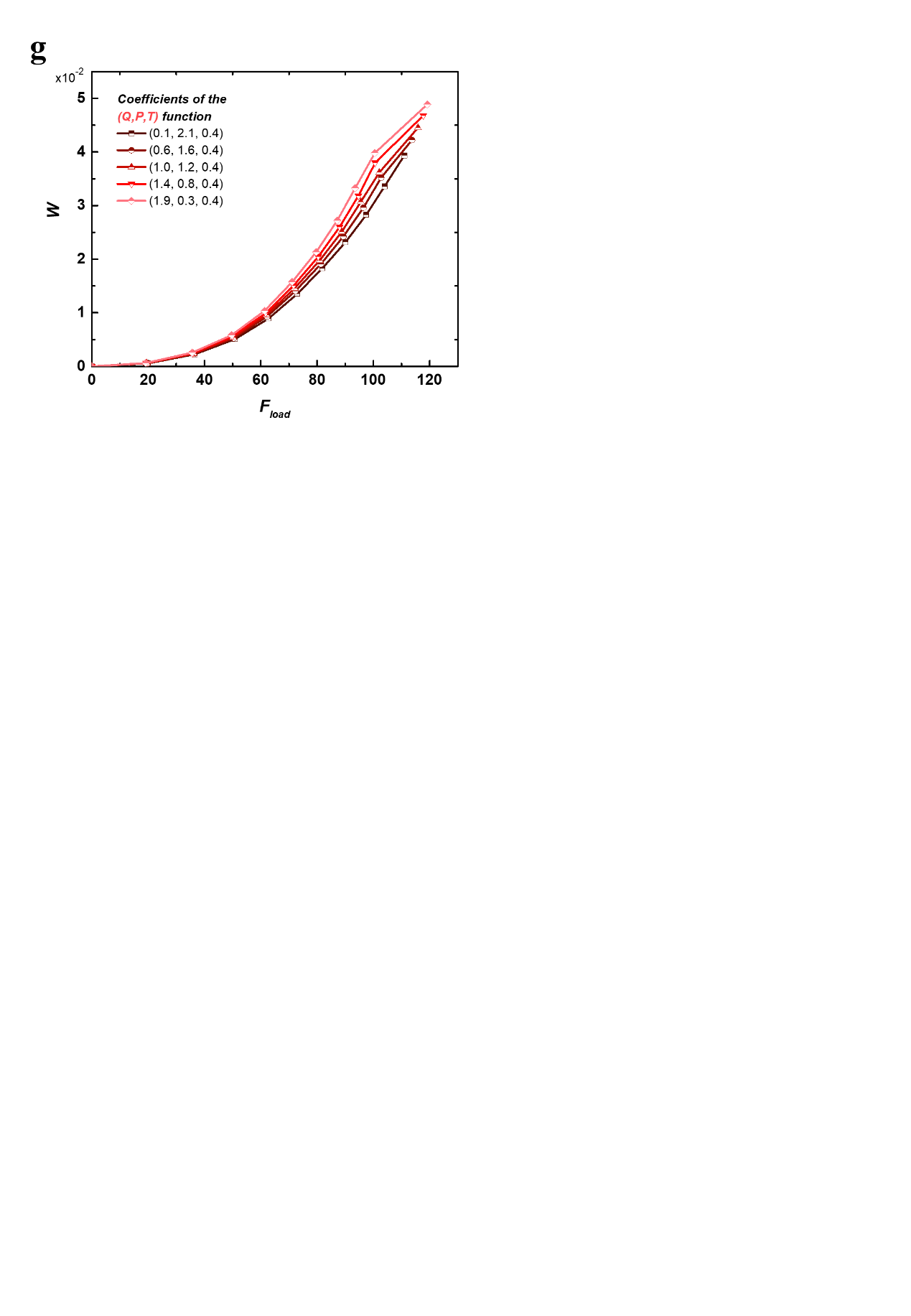


**Supplementary Fig. 9 | a-c.** Vertical (solid line) and horizontal (dashed line) deformation of the T-cell nucleus under individual loads *P* in **a**, *Q* in **b**, and *T* in **c** (normalized). Load distributions are represented by cosine, linear, and constant functions, denoted by different colored curves in each plot. The qualitative relationship between nuclear deformation and forms of the load. **d-e.** Representative deformation of the T-cell nucleus under the combined loads of *P*, *Q*, and *T* in **d**, and under the individual load of *T* in **e.** **f-g.** Vertical deformation of the T-cell nucleus under different load combinations in **f** and strain energy in **g**, with data normalized for comparison.

Fig. S10.


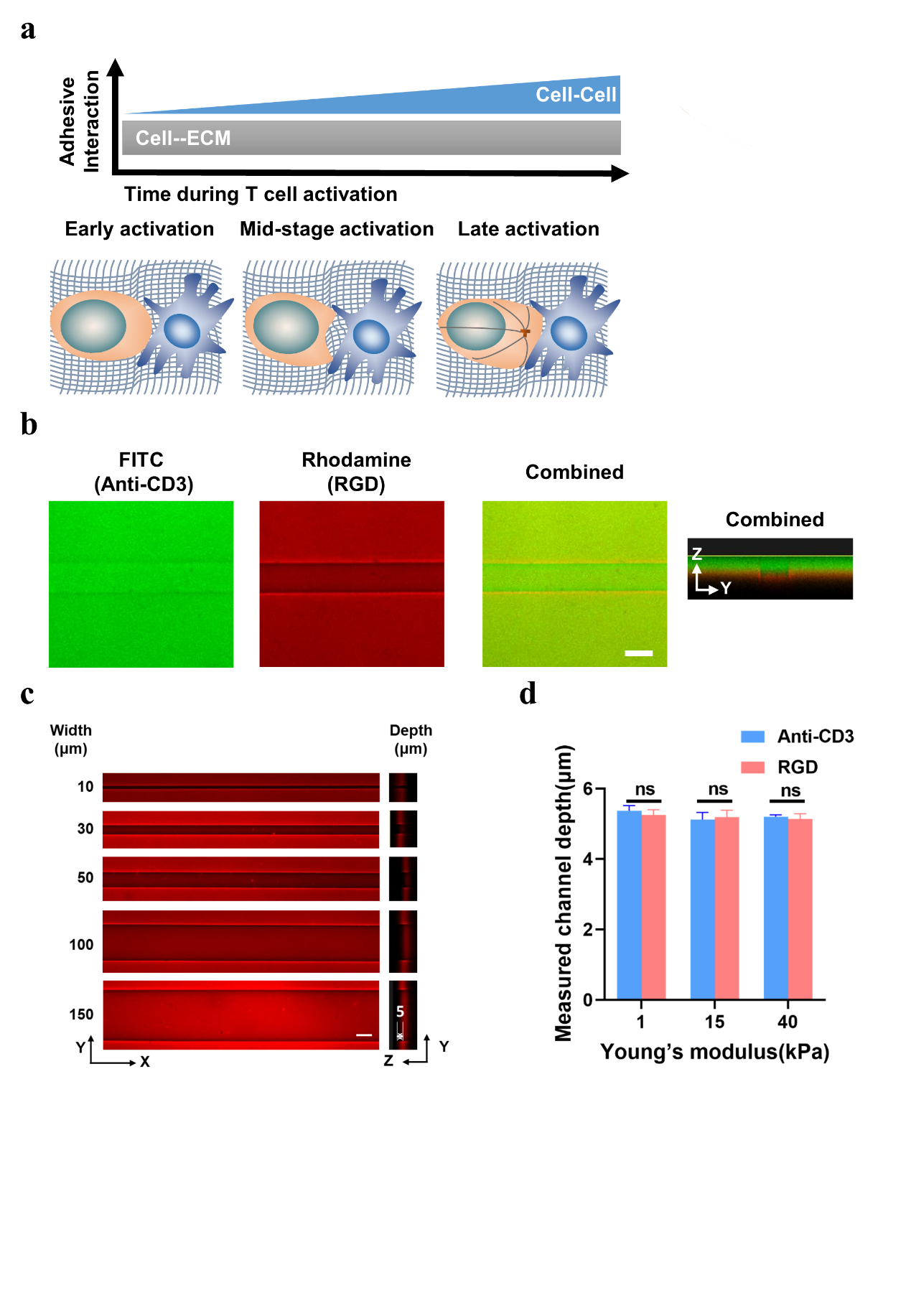


**Supplementary Fig. 10 | a.** Schematic of the evolution of the mechanical microenvironment during T-cell activation for the dynamic mechanical forces were transduced by T cell-ECM interactions, while T cell-APC interactions were persistently present. **b.** Fluorescence images to characterize the distance between two layers of the microchannel PEG hydrogel system in the Z-axis. The anti-CD3 monoclonal antibody coupled with FITC was modified in the lid of the microchannel (green fluorescence), the RGD peptide coupled with rhodamine was modified in the bottom of the microchannel (red fluorescence), and the distance was showed by image reconstruction with laser scanning confocal microscopy. **c.** Representative fluorescence images at the cross-sectional side view were captured along the XY- and XZ-plane for the width and depth of the microchannel PEG hydrogels, which were modified with rhodamine-coupled RGD peptide. **d.** Quantification of the depth of fabricated hydrogel microchannels. (Anti-CD3) represents Anti-CD3 was modified at the bottom with tunable stiffness of the microchannel, (RGD) represents RGD was modified at the bottom with tunable stiffness of the microchannel. Data are presented as mean ±  S.E.M., and *P*-values were obtained using one-way ANOVA followed by Tukey’s post hoc test (c). Scale bars: 50 µm.

Fig. S11.


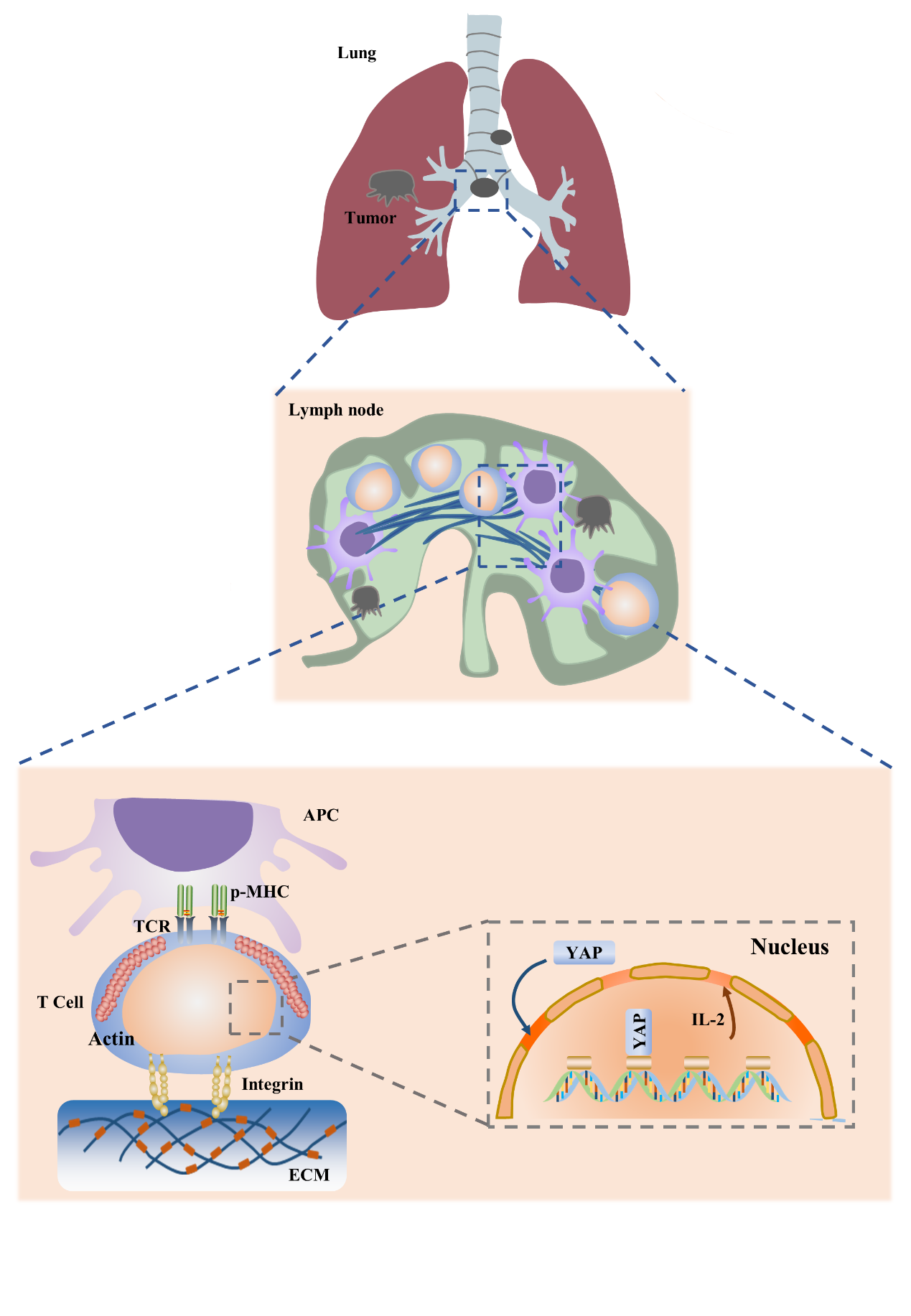


**Supplementary Fig. 11** Schematic of the strategy for studying the mechanotransduction of lymph nodes matrix on T-cell activation.

Supplementary Text

**Stiffness-associated transcriptional scores in lymph nodes**

To corroborate the findings of LN stiffness, we analyzed single-cell RNA-seq data from the publicly available GSE131907 dataset [1], which includes 10 normal lymph nodes (nLN) and 7 metastatic lymph nodes (mLN) from lung adenocarcinoma patients. Processed expression matrices were obtained directly from the GEO database.

To evaluate lymph node stiffness, we defined a stiffness-associated gene set comprising (1) ECM structural components (e.g., COL1A1, COL1A2), (2) integrin signaling molecules (ITGA7, ITGB1, ITGB3, ITGB7), (3) mechanosensitive regulators (YAP1, TRPV4, EPHA2, UBTD1), and (4) fibrosis mediators (TGFB1, THBS1, RASSF1), based on published mechanobiology studies [2-4]. Stiffness scores for individual cells were calculated using the AddModuleScore function in the Seurat (v4.4.0) R package following the official implementation guide (https://satijalab.org/seurat/reference/AddModuleScore). Statistical comparisons between nLN and mLN groups were performed using two-tailed Student’s *t*-test. Subsequent Gene Ontology (GO) enrichment analysis of differentially expressed genes was conducted using the clusterProfiler R package to identify pathways related to ECM remodeling and stromal activation.

**Isolation of primary murine CD8⁺ T cells**

Primary murine CD8⁺ T cells were isolated from spleens using a negative selection kit (Selleck, B90011). Briefly, spleens were mechanically dissociated through a 70 μm cell strainer to obtain single-cell suspensions, followed by red blood cell lysis with ACK buffer. After washing and counting, cells were resuspended in sorting buffer (PBS with 2 mM EDTA and 2% FBS) and sequentially incubated with Biotin-Ab Mix and Streptavidin beads at 4 °C. Labeled cells were removed using a magnetic rack, and the unlabeled CD8⁺ T cells in the supernatant were collected, washed, and resuspended for downstream experiments.

**Simulatrion of Actin Polymerization-Driven T-Cells Nuclear Deformation**

Based on experimental observations (**Fig. 2**, **Fig.4**) and the phenomenon of extensive actin reaggregation induced by T-cell activation [5, 6], we propose a qualitative model to simulate the nuclear deformation process. The actin cytoskeleton of T cells undergoes remodeling, which includes thickening of the cortical actin layer [7], (where its force is decomposed into vertical pressure *P* and tangential force *T* acting on the nuclear surface $s$), as well as the formation of branched actin networks above the immune synapse [8-10] (simplified as vertical pressure *Q* acting on the central region of the nucleus $s'$), shown in **Fig. 5a.**

Experimental observations indicated a significant decrease in the height of the cell nucleus, suggesting that the main direction of force acting on the nucleus is vertically downward. Thus, we assumed that the upper part of the nucleus experiences an active force $\boldsymbol{F}_{load}$ while the lower part is subject to a constraint force $\boldsymbol{F}_{constraint}$ that balances it:

$$\begin{aligned} \sum\boldsymbol{F=}\boldsymbol{F}_{load}\boldsymbol{+}\boldsymbol{F}_{constraint}\boldsymbol{=0}\#(1) \end{aligned}$$

The load acting on the upper part of the nucleus is calculated as:

$$\begin{aligned} \boldsymbol{F}_{load}=\int\left( \boldsymbol{P+T} \right)d\boldsymbol{s}+\int\boldsymbol{Q}d\boldsymbol{s'}\#(2) \end{aligned}$$

The T-cell nucleus was modeled as a two-dimensional shell structure with a diameter of *R* and thickness of *d*, characterized by Young's modulus *E* and Poisson's ratio $\upsilon$.The strain tensor $\boldsymbol{\epsilon}_{ij}$is given by :

$$\begin{aligned} \boldsymbol{\epsilon}_{ij}=\frac{1}{2}\left[ (\nabla\boldsymbol{u}\boldsymbol{)}^{T}+\nabla\boldsymbol{u+}(\nabla\boldsymbol{u}\boldsymbol{)}^{T}\nabla\boldsymbol{u} \right]\#(3) \end{aligned}$$

where $\nabla$ is the Laplace Operator and $\boldsymbol{u}$ is the total displacement. The elastic strain energy *W* of the deformed nucleus is given by :

$$\begin{aligned} W=\int\boldsymbol{\sigma}d\boldsymbol{\varepsilon}\#(4) \end{aligned}$$

Where the $\boldsymbol{\sigma}$ is the stress and $\boldsymbol{\varepsilon}$ is the strain.

The accurate quantification of the load and its distribution on the T-cells nucleus presents challenges. To address this, common mathematical functions, such as trigonometric or linear functions, were selected to simulate the actual load distribution. The selected test functions were $P_{1}=F_{\mathrm{load}}\cos\left( \frac{\pi x}{2R} \right)$, $P_{2}=F_{\mathrm{load}}x/R$, $P_{3}=F_{load}/2$, $T_{1}={\mu F}_{\mathrm{load}}\cos\left( \frac{\pi x}{2R} \right)$, $T_{2}={\mu F}_{\mathrm{load}}x/R$, $T_{3}=\mu F_{\mathrm{load}}/2$, $Q_{1}=F_{\mathrm{load}}\sin\left( \frac{\pi x}{2R\sin\left( \frac{2s}{\pi R} \right)} \right)$, $Q_{2}=\frac{F_{\mathrm{load}}x}{R\sin\left( \frac{2s}{\pi R} \right)}$, and $Q_{3}=F_{\mathrm{load}}/2$. The results demonstrate that the type of function does not significantly affect the displacement-load curves(**Figs. S9a–c**). Therefore, in subsequent simulations, the mean values of the three types of functions were used as the load functions for *P*, *T* and *Q*.

To facilitate the analysis of critical factors, non-dimensionalization was performed on force, displacement, and strain energy. $F_{load}=\mathrm{sum}(P,Q,T)/(E\pi R^{2})$ was employed for force, while $U$ or $U_{Z}=d\mathrm{isplacemen}t/R$ was utilized for max displacement, and $W=strain energy/(FR)$ was adopted for strain energy.

Table S1.

**Model parameters**

| **Symbol** | **Parameter Description** | **Value** | **Ref.** | |
| --- | --- | --- | --- | --- |
| $R$ | Radius of the nucleus | 3μm | | [10] |
| $d$ | Thickness of the nucleus | 0.3 μm | | [10], Adjusted |
| $E$ | Young's modulus of the nucleus | 60 kPa | | [10] |
| $\upsilon$ | Poisson's ratio of the nucleus | 0.4 | | [6, 10] |
| $s$ | The action length of the load *P*,*T* | 9.42 μm | | Adjusted |
| $s'$ | The action length of the load *Q* | 2.83 μm | | Adjusted |
| $F_{load}$ | The sum of the dimensionless forces applied to the nucleus | 0~200 | | [10], Adjusted |

# Reference

1. Kim N, Kim HK, Lee K, Hong Y, Cho JH, Choi JW, Lee JI, Suh YL, Ku BM, Eum HH, et al. Single-cell RNA sequencing demonstrates the molecular and cellular reprogramming of metastatic lung adenocarcinoma*.* *Nat Commun* 2020;11(1):2285.

2. Zhang L, Cao B, Hou Y, Wei Q, Ou R, Zhao B, Shang H. High neutrophil-to-lymphocyte ratio predicts short survival in multiple system atrophy*.* *NPJ Parkinsons Dis* 2022;8(1):11.

3. Yagi M, Ji F, Charlton J, Cristea S, Messemer K, Horwitz N, Di Stefano B, Tsopoulidis N, Hoetker MS, Huebner AJ, et al. Dissecting dual roles of MyoD during lineage conversion to mature myocytes and myogenic stem cells*.* *Genes Dev* 2021;35(17-18):1209-1228.

4. Takashima M, Manabe RI, Ohkuma M. Draft Genome Sequence of Oleaginous Yeast Saitozyma sp. Strain JCM 24511, Isolated from Soil on Iriomote Island, Okinawa, Japan*.* *Microbiol Resour Announc* 2020;9(48).

5. Gupta S, Marcel N, Sarin A, Shivashankar GV. Role of actin dependent nuclear deformation in regulating early gene expression*.* *PLoS One* 2012;7(12):e53031.

6. Gerbal F, Chaikin P, Rabin Y, Prost J. An elastic analysis of Listeria monocytogenes propulsion*.* *Biophys J* 2000;79(5):2259-75.

7. Tsopoulidis N, Kaw S, Laketa V, Kutscheidt S, Baarlink C, Stolp B, Grosse R, Fackler OT. T cell receptor-triggered nuclear actin network formation drives CD4(+) T cell effector functions*.* *Sci Immunol* 2019;4(31).

8. Gonzalez-Granado JM, Silvestre-Roig C, Rocha-Perugini V, Trigueros-Motos L, Cibrian D, Morlino G, Blanco-Berrocal M, Osorio FG, Freije JMP, Lopez-Otin C, et al. Nuclear envelope lamin-A couples actin dynamics with immunological synapse architecture and T cell activation*.* *Sci Signal* 2014;7(322):ra37.

9. Fritzsche M, Fernandes RA, Chang VT, Colin-York H, Clausen MP, Felce JH, Galiani S, Erlenkamper C, Santos AM, Heddleston JM, et al. Cytoskeletal actin dynamics shape a ramifying actin network underpinning immunological synapse formation*.* *Sci Adv* 2017;3(6):e1603032.

10. Fabrikant G, Gupta S, Shivashankar GV, Kozlov MM. Model of T-cell nuclear deformation by the cortical actin layer*.* *Biophys J* 2013;105(6):1316-23.
