# Supplementary figures and images for: Integrin-Mediated Mechanosensing of Modeled Lymph Node Microenvironment Promotes T Cell Activation via Nuclear Deformation
Source: Research (Wash D C). 2026 Feb 6;9:1121. doi: 10.34133/research.1121 (PMC12877341; doi:10.34133/research.1121)

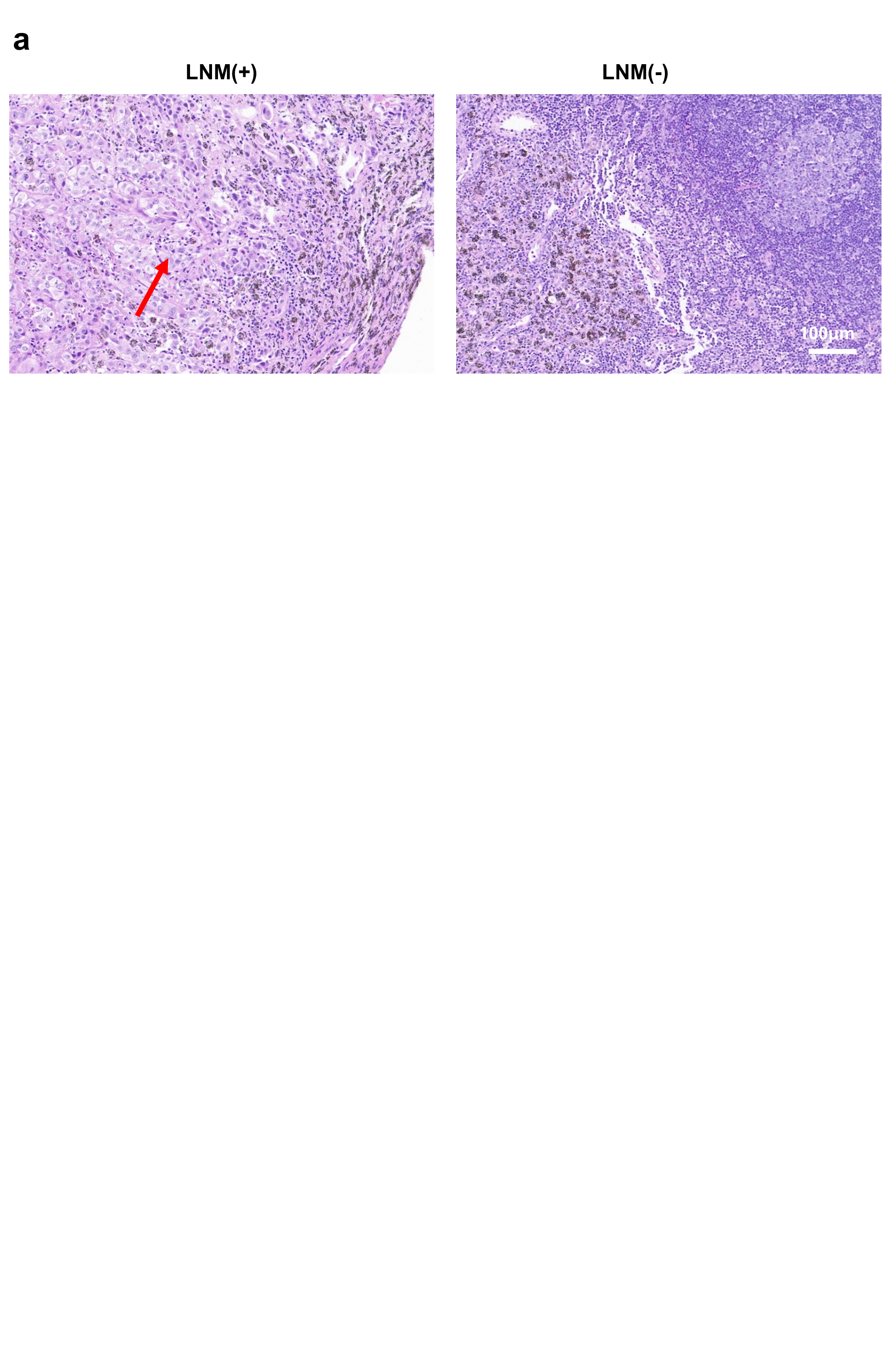

Supplement: Supplementary 1 — Figs. S1 to S11 Supplementary Text Table S1 [file research.1121.f1.zip › Figure S1.TIF]

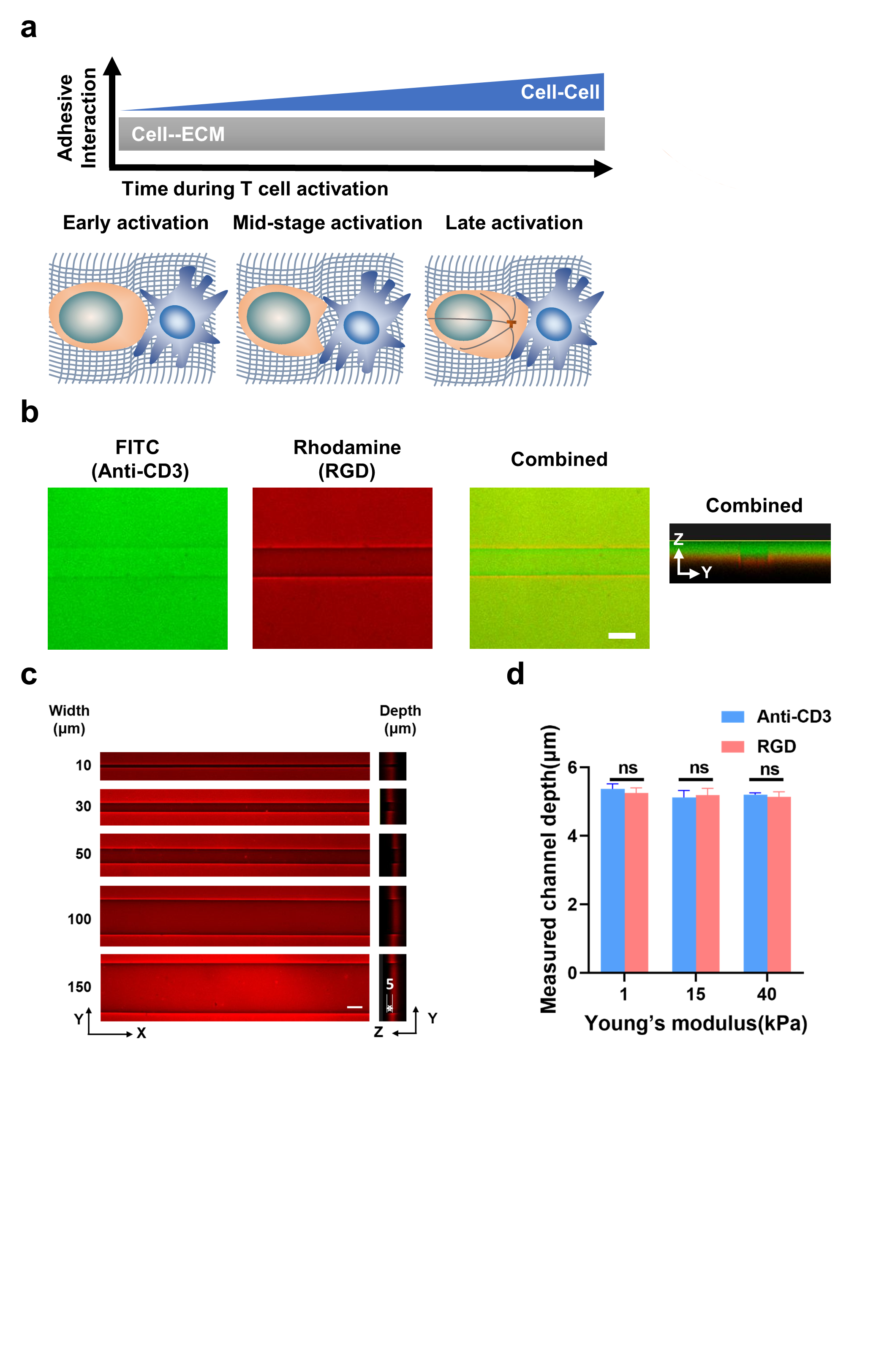

Supplement: Supplementary 1 — Figs. S1 to S11 Supplementary Text Table S1 [file research.1121.f1.zip › Figure S10.TIF]

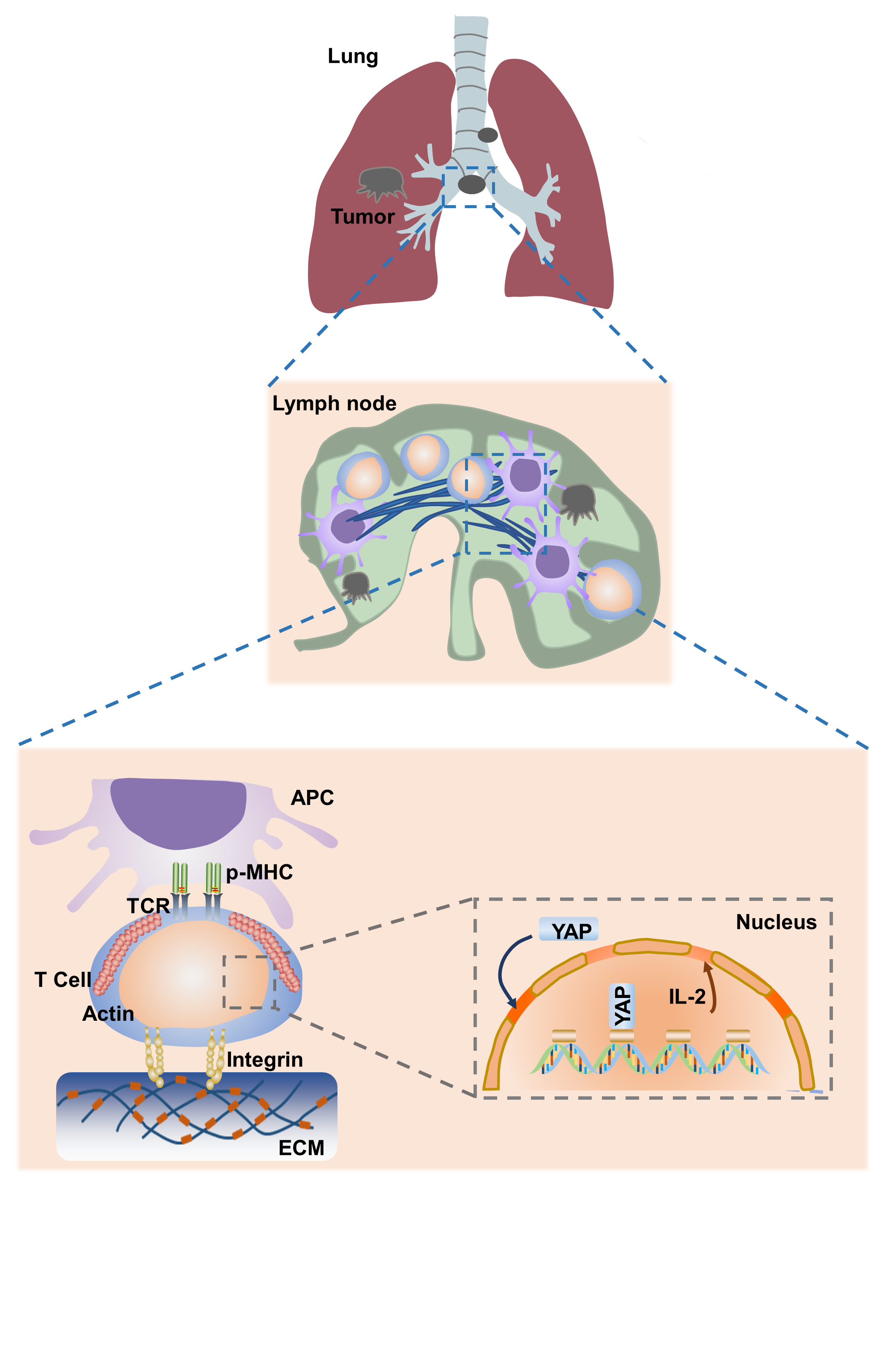

Supplement: Supplementary 1 — Figs. S1 to S11 Supplementary Text Table S1 [file research.1121.f1.zip › Figure S11.TIF]

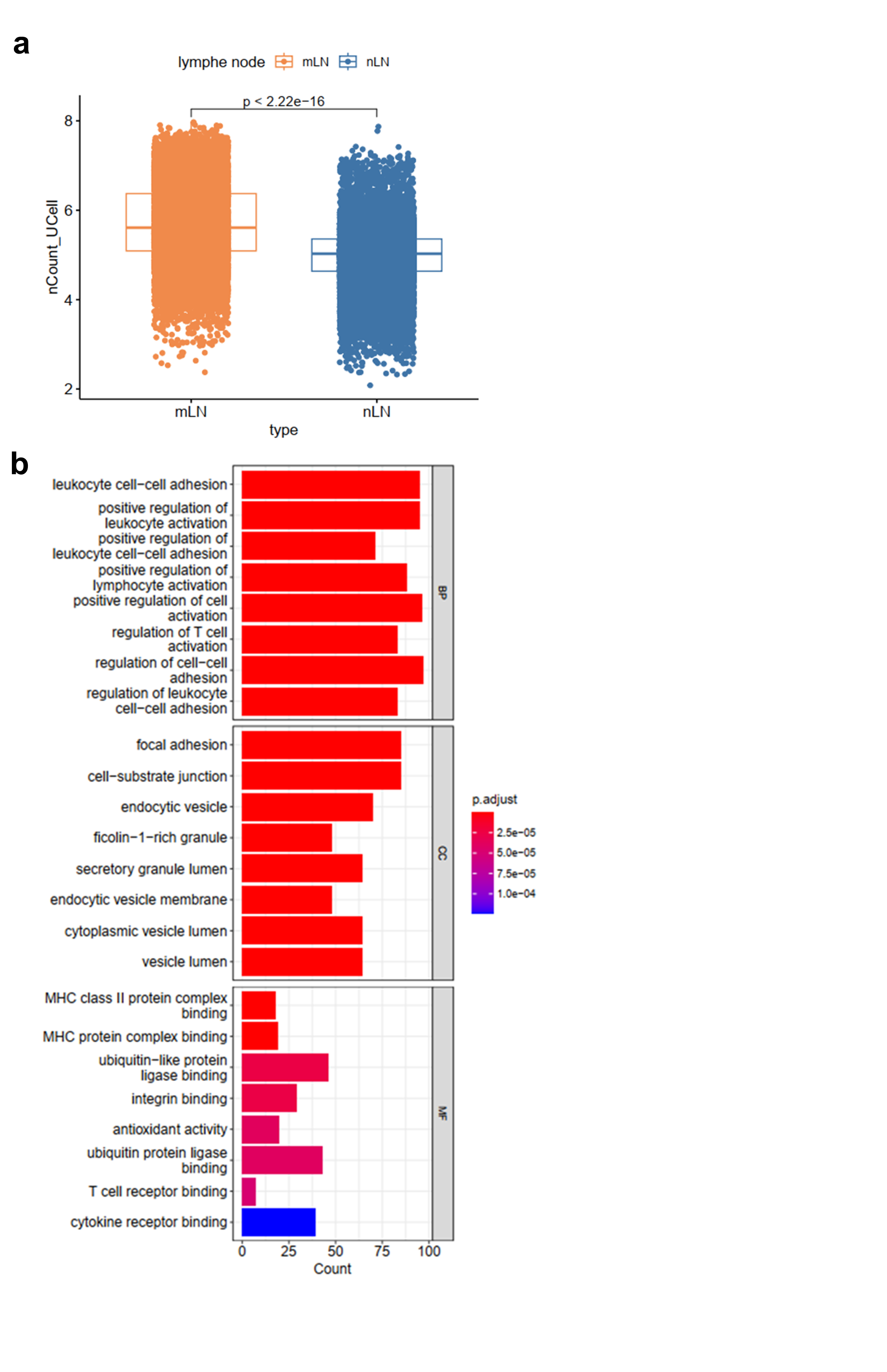

Supplement: Supplementary 1 — Figs. S1 to S11 Supplementary Text Table S1 [file research.1121.f1.zip › Figure S2.TIF]

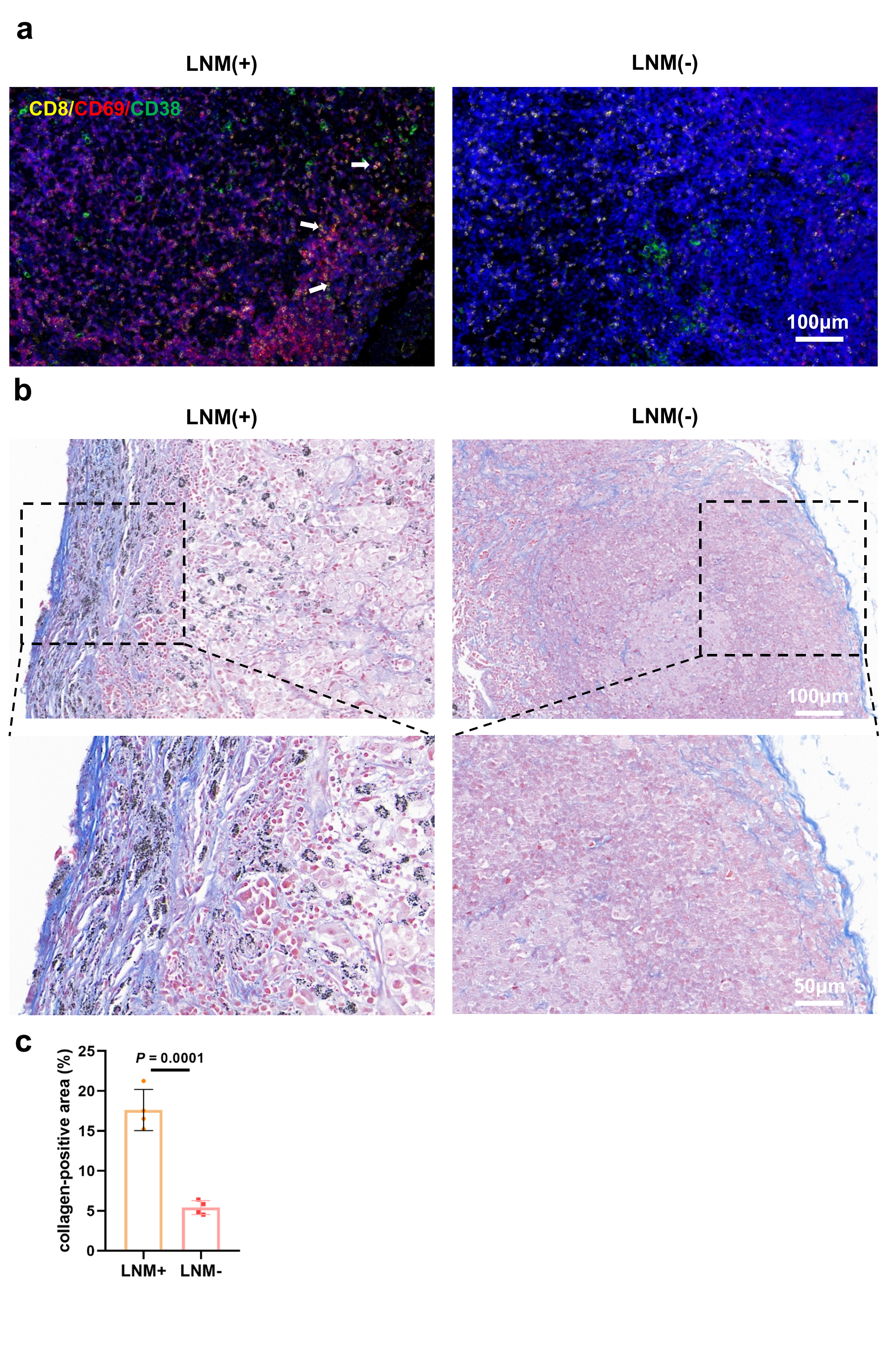

Supplement: Supplementary 1 — Figs. S1 to S11 Supplementary Text Table S1 [file research.1121.f1.zip › Figure S3.TIF]

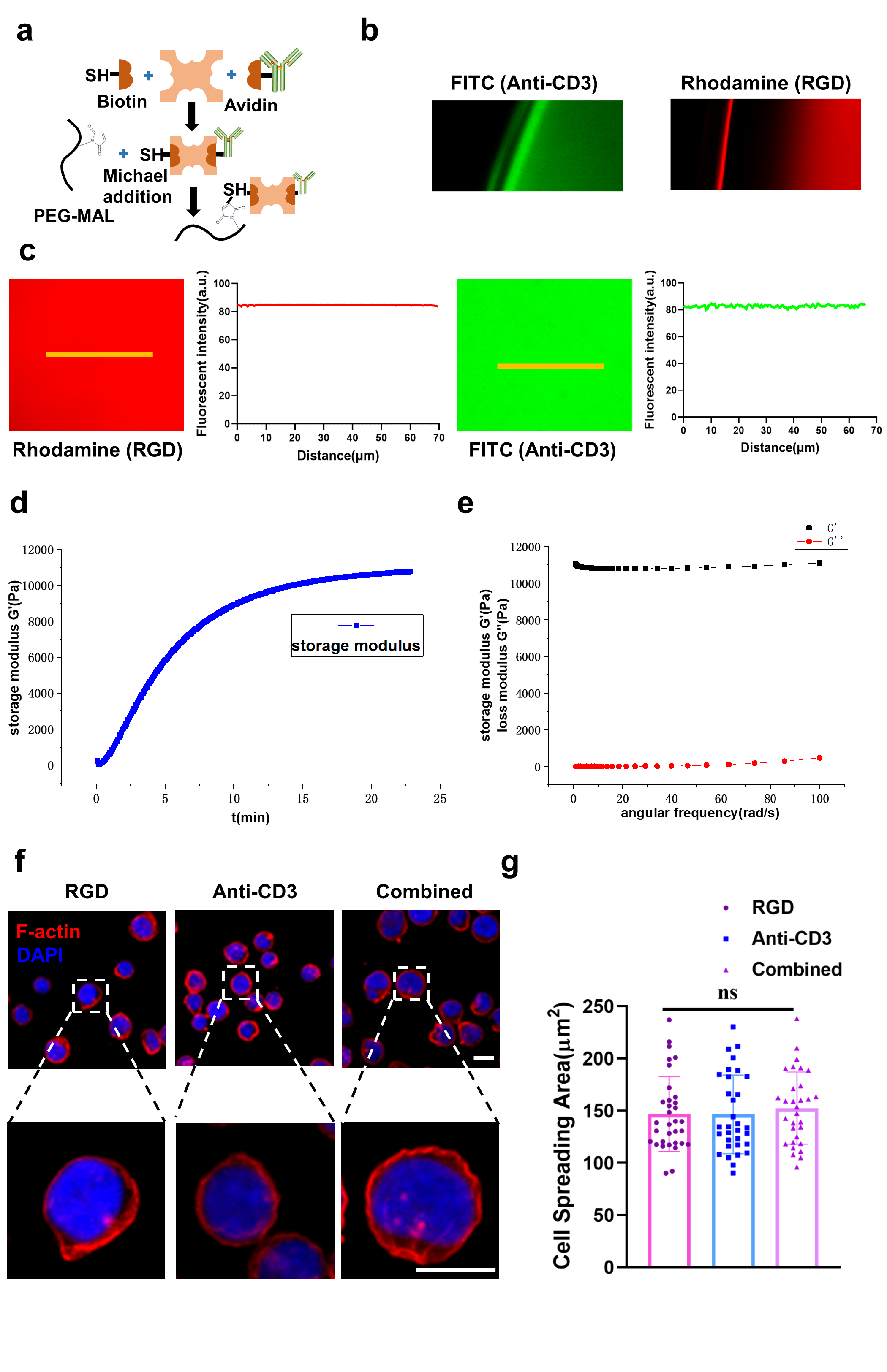

Supplement: Supplementary 1 — Figs. S1 to S11 Supplementary Text Table S1 [file research.1121.f1.zip › Figure S4.TIF]

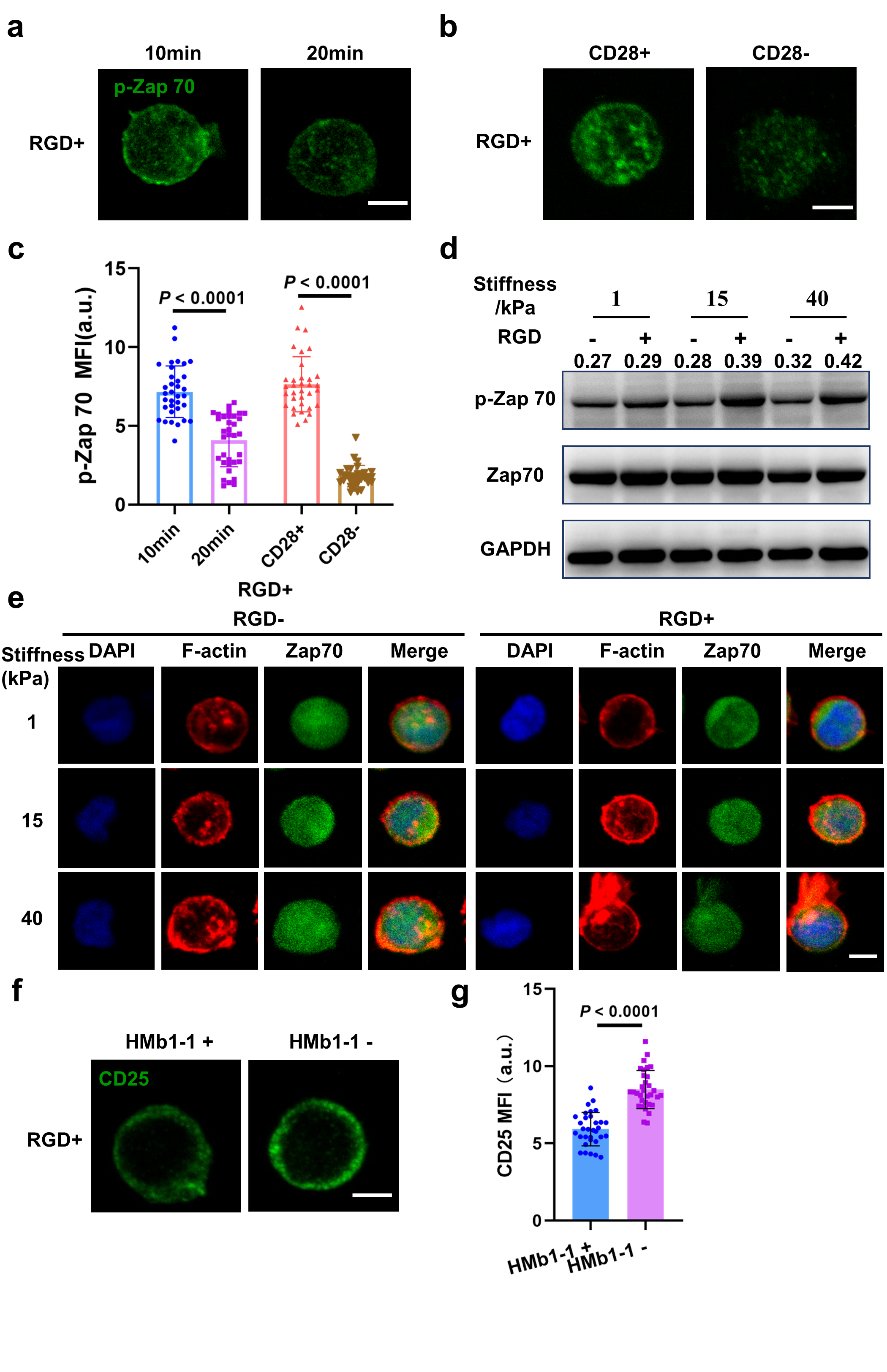

Supplement: Supplementary 1 — Figs. S1 to S11 Supplementary Text Table S1 [file research.1121.f1.zip › Figure S5.TIF]

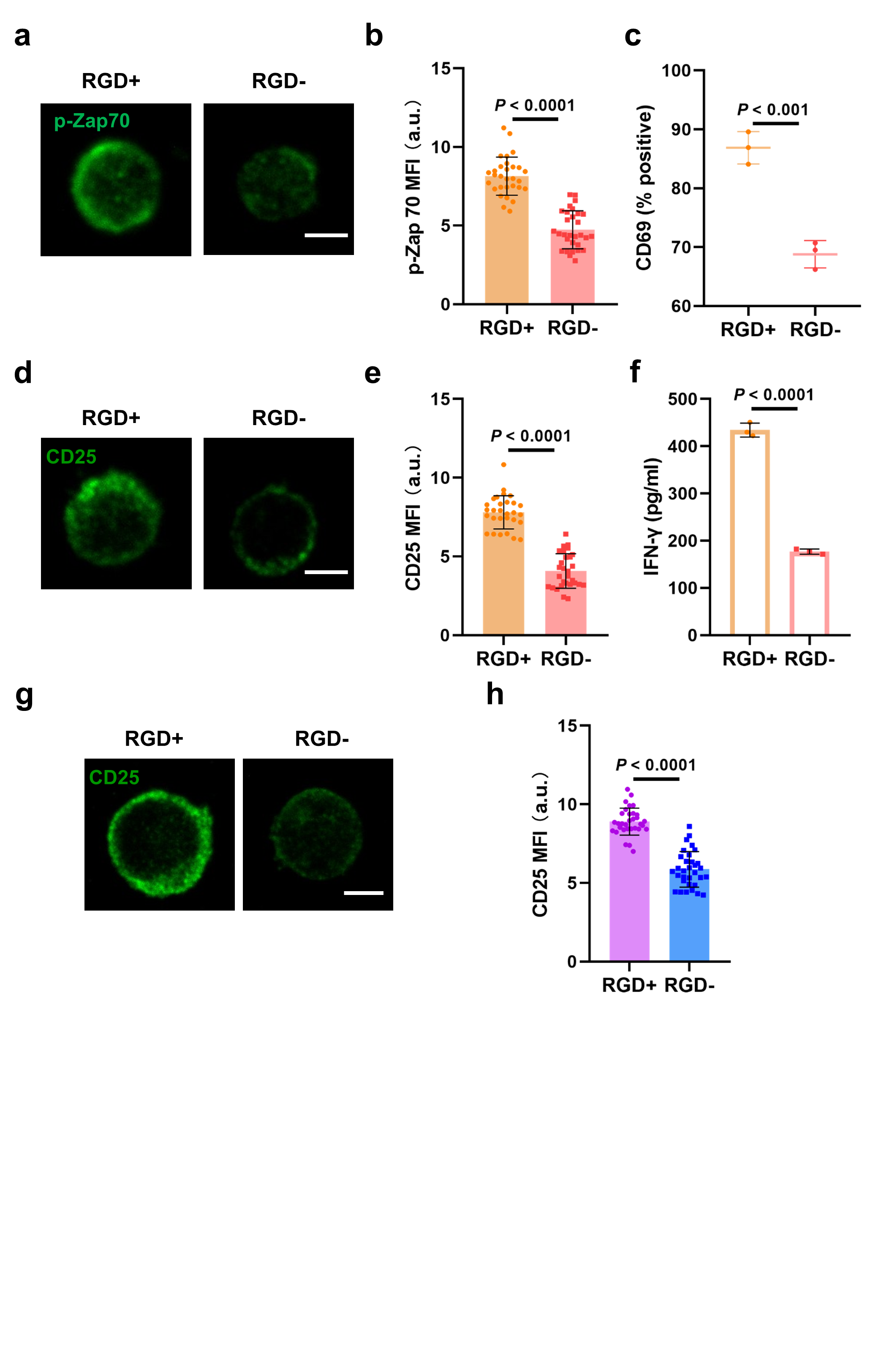

Supplement: Supplementary 1 — Figs. S1 to S11 Supplementary Text Table S1 [file research.1121.f1.zip › Figure S6.TIF]

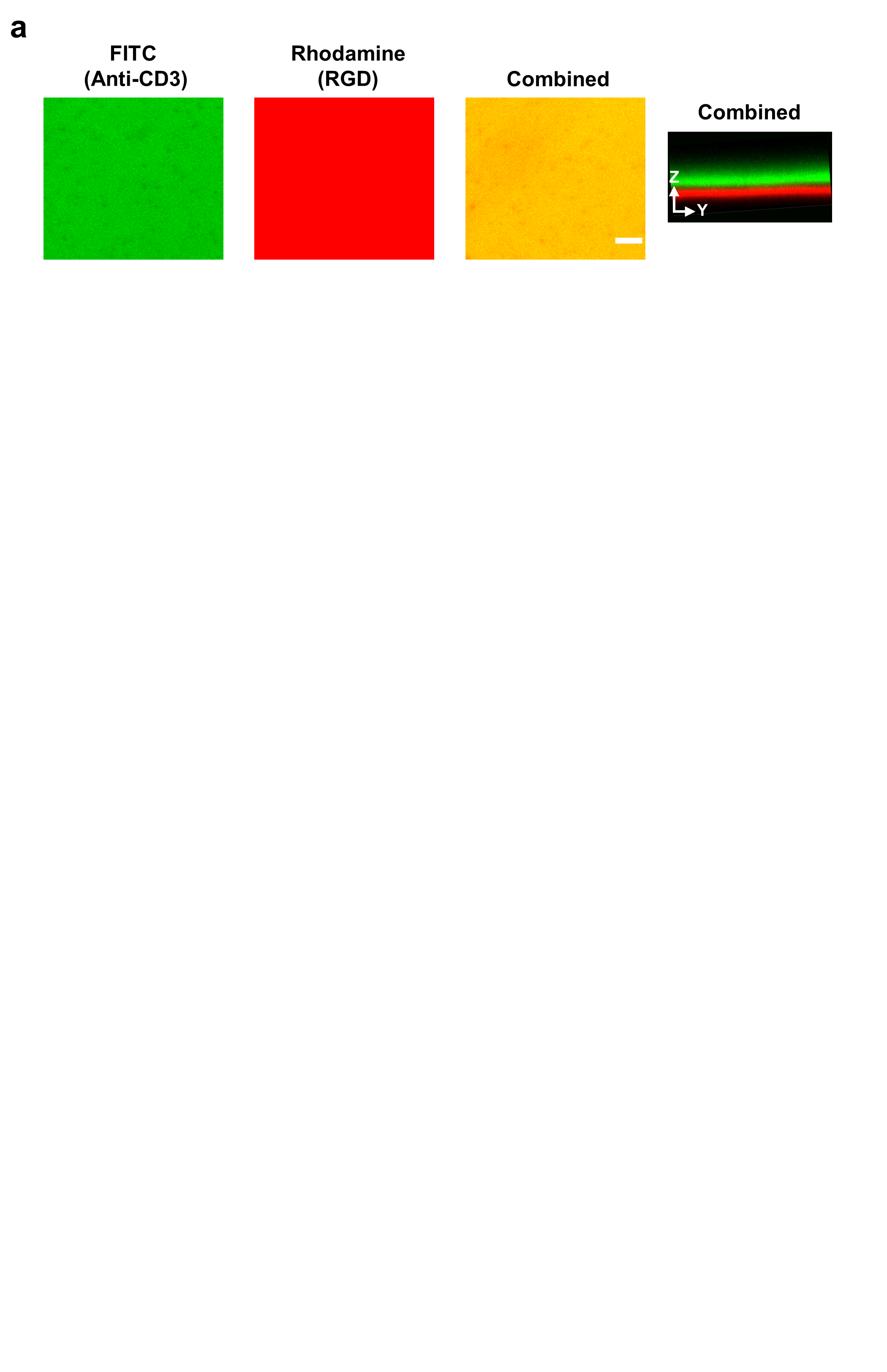

Supplement: Supplementary 1 — Figs. S1 to S11 Supplementary Text Table S1 [file research.1121.f1.zip › Figure S7.TIF]

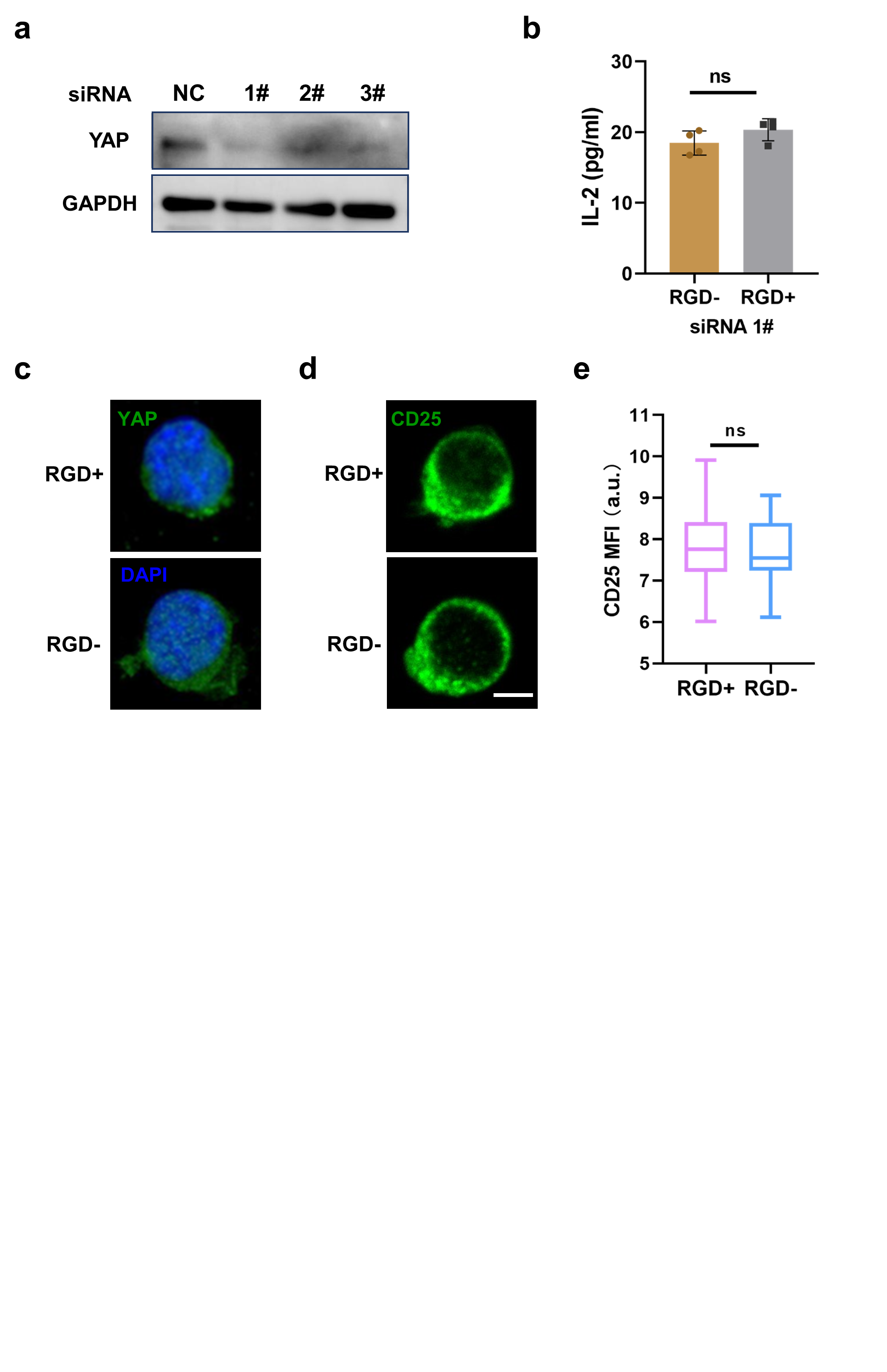

Supplement: Supplementary 1 — Figs. S1 to S11 Supplementary Text Table S1 [file research.1121.f1.zip › Figure S8.TIF]

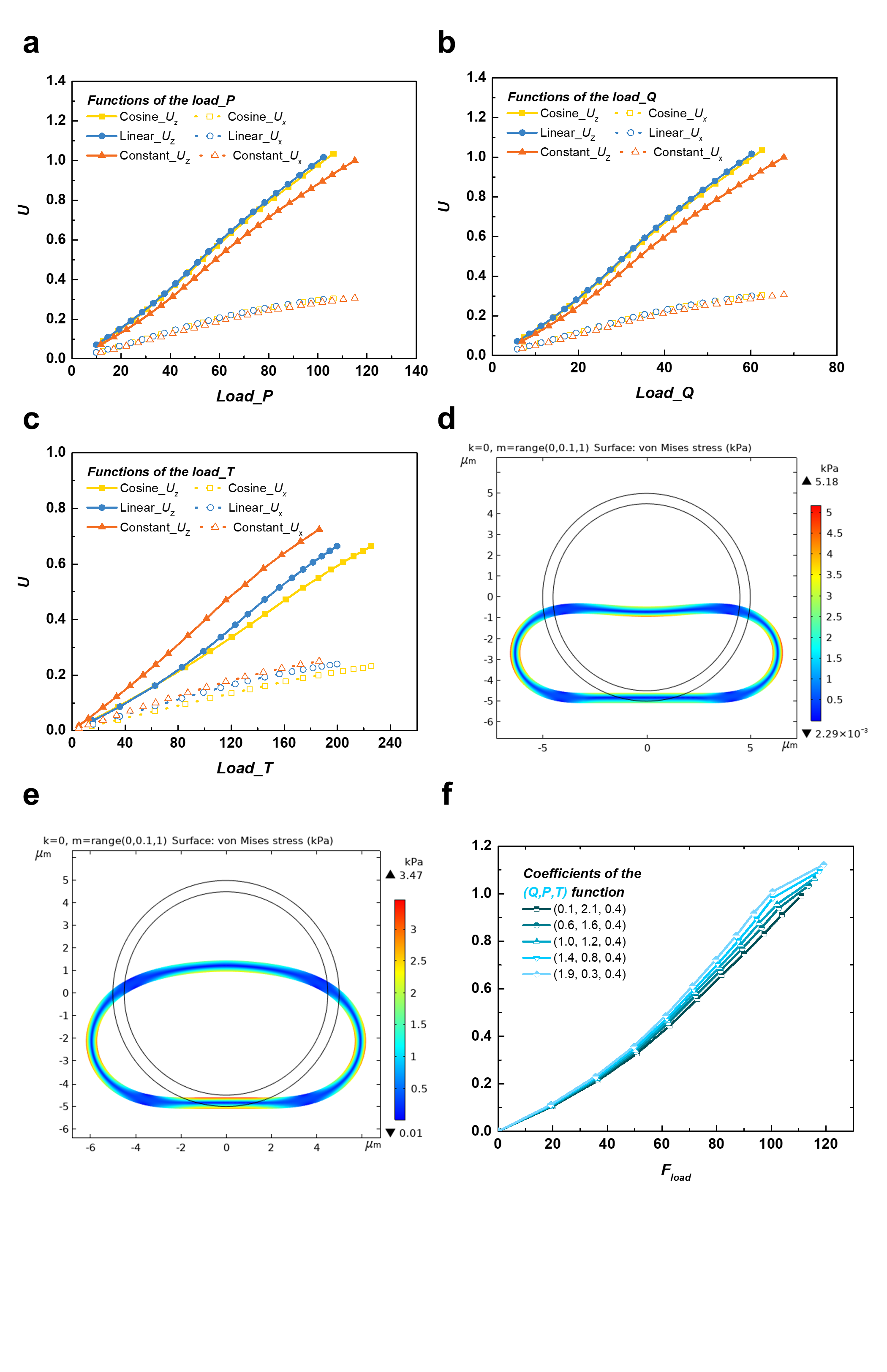

Supplement: Supplementary 1 — Figs. S1 to S11 Supplementary Text Table S1 [file research.1121.f1.zip › Figure S9a-f.TIF]

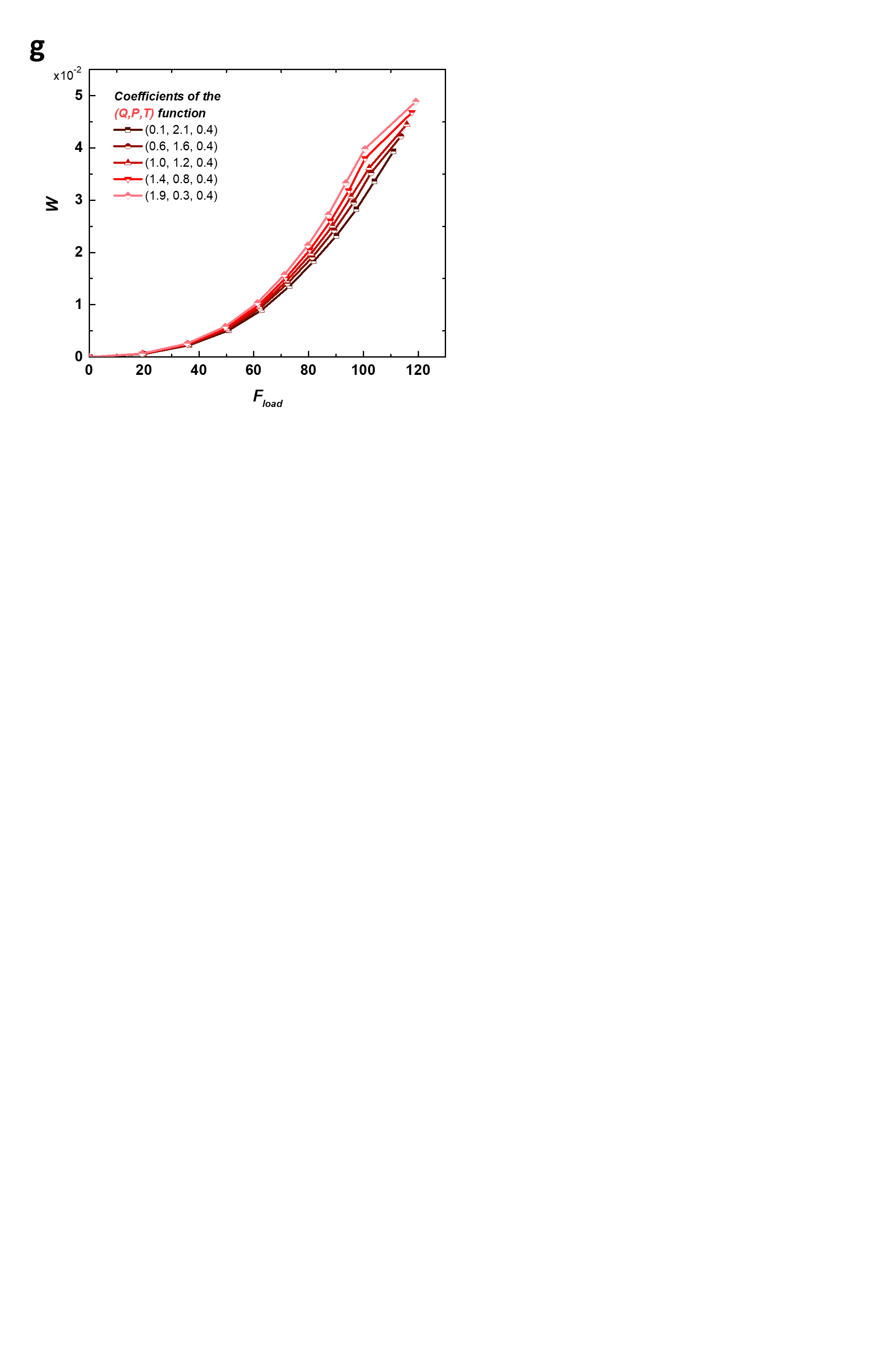

Supplement: Supplementary 1 — Figs. S1 to S11 Supplementary Text Table S1 [file research.1121.f1.zip › Figure S9g.TIF]
